# Dopamine error signal to actively cope with lack of expected reward

**DOI:** 10.1126/sciadv.ade5420

**Published:** 2023-03-10

**Authors:** Seiya Ishino, Taisuke Kamada, Gideon A. Sarpong, Julia Kitano, Reo Tsukasa, Hisa Mukohira, Fangmiao Sun, Yulong Li, Kenta Kobayashi, Honda Naoki, Naoya Oishi, Masaaki Ogawa

**Affiliations:** ^1^ Medical Innovation Center/SK Project, Graduate School of Medicine, Kyoto University, Kyoto 606-8507, Japan.; ^2^ Department of Neuroscience, Graduate School of Medicine, Kyoto University, Kyoto 606-8501, Japan.; ^3^ Department of Developmental Physiology, National Institute for Physiological Sciences, Okazaki, Aichi 444-8585, Japan.; ^4^ State Key Laboratory of Membrane Biology, Peking University School of Life Sciences, Beijing 100871, China.; ^5^ Peking-Tsinghua Center for Life Sciences, Beijing 100871, China.; ^6^ PKU-IDG/McGovern Institute for Brain Research, Beijing 100871, China.; ^7^ Section of Viral Vector Development, National Institute for Physiological Sciences, Okazaki, Aichi 444-8585, Japan.; ^8^ SOKENDAI (The Graduate University for Advanced Studies), Okazaki, Aichi 444-8585, Japan.; ^9^ Laboratory of Data-driven Biology, Graduate School of Integrated Sciences for Life, Hiroshima University, Higashi-Hiroshima, Hiroshima 739-8526, Japan.; ^10^ Theoretical Biology Research Group, Exploratory Research Center on Life and Living Systems (ExCELLS), National Institutes of Natural Sciences, Okazaki, Aichi 444-8787, Japan.; ^11^ Laboratory of Theoretical Biology, Graduate School of Biostudies, Kyoto University, Kyoto 606-8315, Japan.; ^12^ Kansei-Brain Informatics Group, Center for Brain, Mind and Kansei Sciences Research (BMK Center), Hiroshima University, Kasumi, Minami-ku, Hiroshima 734-8551, Japan.

## Abstract

To obtain more of a particular uncertain reward, animals must learn to actively overcome the lack of reward and adjust behavior to obtain it again. The neural mechanisms underlying such coping with reward omission remain unclear. Here, we developed a task in rats to monitor active behavioral switch toward the next reward after no reward. We found that some dopamine neurons in the ventral tegmental area exhibited increased responses to unexpected reward omission and decreased responses to unexpected reward, following the opposite responses of the well-known dopamine neurons that signal reward prediction error (RPE). The dopamine increase reflected in the nucleus accumbens correlated with behavioral adjustment to actively overcome unexpected no reward. We propose that these responses signal error to actively cope with lack of expected reward. The dopamine error signal thus cooperates with the RPE signal, enabling adaptive and robust pursuit of uncertain reward to ultimately obtain more reward.

## Teaser

A dopamine circuit provides a mechanism for adaptive reward pursuit to overcome lack of reward and ultimately obtain more reward.

## INTRODUCTION

Rewards are often uncertain and not easily obtained. When we need to continue to pursue a particular uncertain reward, how do we achieve the goal of obtaining more of that reward? Even if the expected reward is not obtained and the negative outcome is disappointing, we should not give up on obtaining the reward. Rather, we need to learn to actively overcome the lack of expected reward and adjust behavior to obtain it again. This ability to cope with lack of expected reward is the key to pursue uncertain rewards and ultimately obtain more rewards. Deficiency in this ability may lead to depressive states, whereas excessive pursuit of a particular target despite negative consequences may lead to addiction. In animal behavior, failure to actively cope with negative outcomes in foraging and courtship behaviors, which are often uncertain and have limited choice options, affect the survival of the species.

When animals are required to continue to pursue a probabilistic reward by performing a specific action without any choice options, they can actively switch their behaviors toward pursuing the next opportunity to obtain the reward even after it is not presented by chance [e.g., (*1*)]. Furthermore, behaviors that are partially reinforced, such as those rewarded with 50% probability, are known to be more resistant (i.e., slower to stop responding) to extinction than behaviors that are continuously (i.e., 100%) reinforced (*2*–*4*). This effect is called partial reinforcement extinction effect and is the most well-known paradoxical reward effect (*2*–*4*). The effect is paradoxical in terms of reward value because the expected value of 50% reward is less than 100% reward. The ability to pursue the next reward after the lack of reward is not based on instantaneous change in reward value because the expected value of the reward decreases after the no reward (NR). Rather, the ability is thought to be based on the experience of eventually obtaining reward after NR (*2*–*7*). Critically, through learning of the relationship between occasional lack of expected reward and antecedent cues, animals can predict the NR in advance and can cope more adaptively with the negative outcome. Thus, in situations where the likelihood of NR is high, animals can learn to stop engaging in the situation and actively switch toward the next opportunity earlier. This is advantageous in allocating efforts efficiently and ultimately obtaining more reward. However, the neural mechanisms underlying this active and adaptive ability to cope with lack of expected reward are poorly understood. A potential candidate for this ability is the midbrain dopamine (DA) neurons. Midbrain DA neurons are well known to provide error signal for reward receipt, termed reward prediction error (RPE), defined as the discrepancy between obtained reward and expected reward. RPE-type DA neurons are thought to be critical for moment-by-moment computation for values and value-based learning (*8*–*16*). However, the activity of the RPE neurons decreases in the face of unexpected NR, which leads to a decrease in reward value and supports negative learning (*8*–*12*, *14*, *16*). Thus, RPE neurons do not directly support the ability to cope with lack of expected reward and adjust behavior toward the next opportunity to obtain the reward. Furthermore, although recent studies revealed that DA neurons are heterogeneous and signal more than RPE (*12*, *17*–*26*), the role of DA neurons in actively coping with lack of expected reward is unknown.

Here, we developed a task that required head-restrained rats to continue to pursue a probabilistic reward by repeating a specific sequence of actions without any choice options. The task allowed us to monitor active behavioral switching toward the next opportunity to obtain reward after its omission. We combined the task with in vivo single-unit recording and single-cell calcium imaging from DA neurons in the ventral tegmental area (VTA), DA measurement in the nucleus accumbens (NAc), and optogenetics. We found that a subset of DA neurons in lateral VTA and DA levels in a part of the NAc exhibited increased responses to unexpected reward omission and decreased responses to unexpected reward. We interpret that these responses signal error to actively, rather than passively, cope with lack of reward based on the reward expectation. The responses were slower than those of the opposite responses of the RPE-type DA neurons. By further introducing tasks such as reward extinction task and transition to a Pavlovian task, we show that the DA error signal in the NAc primarily provides a mechanism for learning to adjust behavior to actively overcome unexpected NR. Our results demonstrate that the DA error signal to cope with lack of expected reward cooperates with the RPE-type DA error signal, allowing adaptive and robust pursuit of uncertain reward to ultimately obtain more reward.

## RESULTS

### Monitoring of active switching toward the next opportunity to obtain reward after the lack of reward

**Fig. 1.**
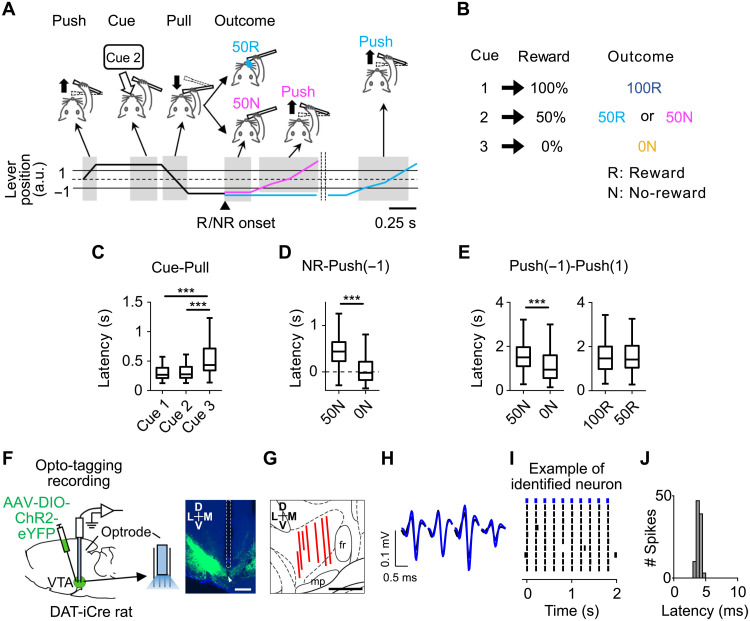
Monitoring of active behavioral switching toward the next opportunity to obtain reward and opto-tagging recording from DA neurons in the VTA. (**A**) Top: Sequence of the behavioral events in the task. “50R,” reward after cue 2 (light blue); “50N,” NR after cue 2 (magenta). Bottom: Trajectory of the lever tip. Reward or NR (R/NR) onset was 0.4 s after “Pull”. a.u., arbitrary unit. (**B**) Relationship between cue and reward probability (left) and outcomes (right). (**C**) Average latency from odor cue offset to pull the lever closer than lever-position(−1) (“Cue-Pull”). *n* = 101 sessions across seven rats. Center lines, median; box limits, 25th and 75th percentiles; whiskers, range. Significant difference between conditions, ***P < 0.001, two-sided Wilcoxon signed-rank test with Bonferroni correction. (**D**) Average latency from NR onset to push the lever beyond the lever-position(−1) [“NR-Push(−1)”]. “0N,” NR after cue 3. ***P < 0.001, two-sided Wilcoxon signed-rank test. (**E**) Average latency from Push(−1) to push forward more than the position(1) [“Push(−1)-Push(1)”] after NR (left) or the last shot of reward presentation (right). ***P < 0.001, two-sided Wilcoxon signed-rank test. (**F**) Left: Schematic of extracellular recording from DA neurons. Right: Example track of an optrode. Green, ChR2-eYFP. Blue, DAPI (4′,6-diamidino-2-phenylindole). Arrowhead, the tip of the tetrode. Scale bars, 500 μm. (**G**) Optrode tracks in the left VTA (*n* = 7 rats) shown on top of the atlas image. Each red line indicates the range of recording locations in each rat projected onto the slice of AP: −5.2 mm. Scale bars, 500 μm. fr, fasciculus retroflexus; mp, mammillary peduncle. (**H**) Example average waveforms of an identified DA neuron recorded from a tetrode (black, waveforms during behavior; blue, light-evoked waveforms). (**I**) Example light-evoked spikes (black tick) to 5-Hz blue-light stimulation (blue tick, top) of an identified DA neuron. (**J**) Histogram of the light-evoked spike latency of the neuron [as in (I)]. Mean = 3.88, SD = 0.34.

To quantitatively measure the ability to actively and adaptively overcome lack of expected reward, we developed an operant task in which rats were required to continue to pursue a probabilistic reward by repeating a specific sequence of actions without any choice options (Fig. 1, A and B).

An illumination of a cue light signaled that a head-restrained rat can self-initiate a trial by pushing forward a spout-lever (“Push”), which triggered a presentation of a 0.3-s odor cue (“Cue”) (Fig. 1A). The cue light was kept on until reward outcomes were presented. One of three odors was presented on each trial in a pseudo-random order, associated with 100, 50, or 0% probability of reward (Fig. 1B). The tip of the lever was equipped with a spout to deliver a water reward (*27*, *28*). After a termination of the odor cue, rats were allowed to pull back the lever toward their mouth (“Pull”), which resulted in a delivery of a reward after a 0.4-s delay or NR (“Outcome”) (Fig. 1A). If rats pulled the lever before the termination of the odor cue, then the trial was regarded as an error trial, and a high-tone sound was presented and the cue light was turned off. After error trials, the same odor was presented in the next trial. These error trials were excluded from the following analysis. The percentage of trials that rats executed correctly without errors was about 80% (fig. S1, A to C).

Rats pulled back the lever in almost all trials including following cue 3 associated with 0% reward (fig. S1D). The latency from cue offset to pull the lever (“Cue-Pull” latency) was slowest after cue 3 associated with 0% reward, suggesting that rats expected a very low reward probability immediately after the presentation of cue 3 (Fig. 1C). The Cue-Pull latency after presentation of cue 2 associated with 50% reward was comparable to cue 1 associated with 100% reward (Fig. 1C) (*1*, *29*) . Critically, although rats could not, in principle, predict which cue would be presented in a given trial, the pseudo-random sequences of the cue presentations can allow rats to expect that if they continue with the task, they would be rewarded in about 50% of all trials. Consistent with the interpretation that the rats learned the task structure across trials, following NR associated with cue 2 (“50N”: expected reward percentage was 50%, and the outcome was NR) and NR associated with cue 3 (“0N”), rats not only stopped the lever-pulling but also pushed the lever forward before the start of the next trial (Fig. 1, A, D, and E). This was also the case after the end of reward delivery (Fig. 1, A and E). The common behavior suggests that rats actively switch their behavior from the current reward pursuit to initiating the pursuit of the next potentially available reward. The latency from NR onset to the lever-pushing [“NR-Push(−1)” and “Push(−1)-Push(1)” latency; Fig. 1, D and E] was slower after unexpected NR after cue 2 (50N) than expected NR after cue 3 (0N) (Fig. 1, D and E). This behavior suggests that after 0N, rats immediately stopped engaging in the current trial and moved on to the next reward, whereas after 50N, rats spent more time checking whether the current reward may be available. Thus, rats adjust how fast they switch toward the next trial according to the expected probability of reward.

### Activity of a subset of DA neurons increase in responses to unexpected NR and decreases in response to unexpected reward

To record the spiking activity of DA neurons in the task, we implemented an “opto-tagging” method that enabled a cell type–specificelectrophysiological recording (*16*, *30*), using *DAT(Slc6a3)-iCre* (DAT-iCre) rats expressing improved Cre recombinase (iCre) under the control of the DA transporter promoter (Fig. 1, F to J, and fig. S2, A and B) (*31*). We recorded single units from the anterior part of lateral VTA in behaving rats (*n* = 7 rats; Fig. 1G) and identified 36 well-isolated DA neurons. We also recorded from putative DA neurons (*n* = 150; fig. S2C). The recording was conducted in relatively late sessions (32.5 sessions on average; see Materials and Methods for details) in which the behavior was stable (Fig. 1, C to E).

**Fig. 2.**
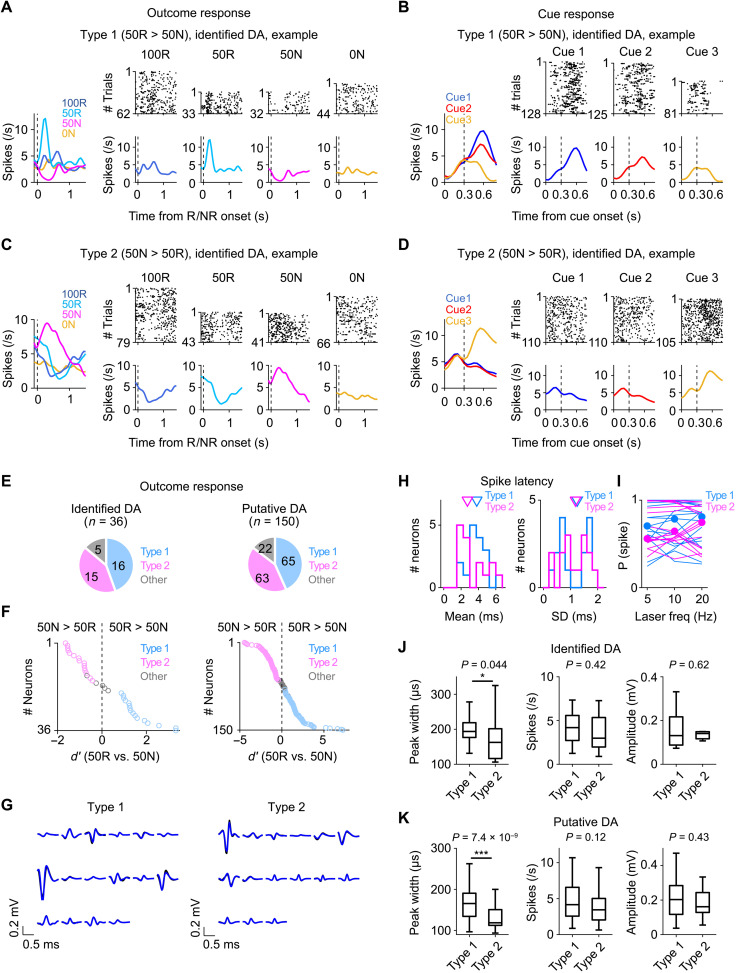
A subset of DA neurons responds to unexpected reward and NR in the opposite direction to RPE neurons. (**A**) Left: Average spiking rates of an example optogenetically identified type 1 DA neuron aligned to outcome onset. Mean across trials. Right: raster plot (top) and average response across all trials (bottom). (**B**) Same as (A) but for cue responses. Dotted line, cue offset. (**C** and **D**) Same as (A) and (B), respectively, but for type 2 DA neuron. (**E**) Number of each type of neuron among identified (left, *n* = 36) or putative (right, *n* = 150) neurons. (**F**) Distribution of *d*′ comparisons of activity to 50R versus 50N of identified (left) or putative (right) neurons. (**G**) Average waveforms of identified type 1 (left, *n* = 16) or type 2 (right, *n* = 15) DA neurons (black, waveforms during behavior; blue, light-evoked waveforms). (**H**) Histogram of mean (left) and SD (right) of spike latency of identified DA neurons in response to the light stimulation (blue: type 1, *n* = 16; magenta: type 2, *n* = 15). No significant difference between the groups. *P* = 0.26 or 0.77, respectively, two-sided Mann-Whitney *U* test. (**I**) Probability of the induction of spikes as a function of stimulation frequency for each neuron (line) and the median across neurons (dot). No significant difference between the groups. *P* = 0.69, two-sided Mann-Whitney *U* test. (**J**) Average peak width (left), spiking rate (middle), or amplitude (right) of identified DA neurons (*n* = 16 or 15 of type 1 or type 2 neurons, respectively). Significant difference between the types, *P = 0.044, two-sided Mann-Whitney *U* test (left). *P* = 0.42 (middle); *P* = 0.62 (right). (**K**) Same as (J) but for putative neurons (*n* = 65 or 63 of type 1 or type 2 neurons, respectively). Significant difference between conditions, ***P = 7.4 × 10^−9^, two-sided Mann-Whitney *U* test (left). *P* = 0.12 (middle); *P* = 0.43 (right).

To examine whether there are DA neurons that might provide error signal in response to unexpected reward or NR, we searched for neurons that showed differential responses to unexpected reward after cue 2 (50% reward: 50R) versus unexpected NR after cue 2 (50N). Among 36 optogenetically identified DA neurons, 16 neurons (44.4%) were more activated by 50R than 50N (“type 1”) (Fig. 2, A, B, and E). By contrast, we found that 15 DA neurons (41.7%) were more activated by 50N than 50R (Fig. 2, C to E) (“type 2”). The difference of the responses to 50R versus 50N in each neuron was continuously distributed (Fig. 2F). These results were also true when we examined putative DA neurons; among 150 neurons, 65 neurons (43.3%) were more activated by 50R, and 63 neurons (42.0%) were more activated by 50N (Fig. 2E). Spiking rates, spike amplitudes, and spike latencies in response to the light stimulation were not different between these two types of DA neurons (Fig. 2, G to K). However, spike width of type 2 neurons was narrower than that of type 1 neurons (Fig. 2, J and K, and fig. S2C).

**Fig. 3.**
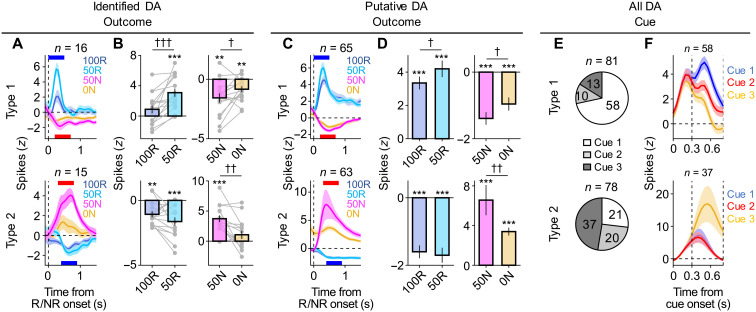
Responses of type 2 DA neurons are opposite to those of RPE neurons, consistent with type 2 error signal. (**A**) Average *z*-scored (*z*) spiking rates of all identified DA neurons during the outcome periods (type 1, *n* = 16; type 2, *n* = 15 neurons). Means ± SEM. across neurons. Blue bar, time window to compare responses to 50R versus 100R (0 to 0.5 s for type 1; 0.4 to 0.9 s for type 2) [as in (B)]. Red bar, time window to compare responses to 50N versus 0N (0.2 to 0.7 s for type 1; 0.3 to 0.8 s for type 2) [as in (B)]. (**B**) Comparisons of average activity (*z*-scored) of the type 1 (top) or type 2 (bottom) neurons during the outcome periods [time windows as in (A)]. Bar graph, mean ± SEM. Gray dot and line, each neuron. Left: In response to 100R versus 50R. Right: In response to 50N versus 0N. Significant difference from baseline, **P < 0.01; ***P < 0.001, two-sided Wilcoxon signed-rank test. Significant difference between conditions, †*P* < 0.05, ††*P* < 0.01, and †††*P* < 0.001, two-sided Wilcoxon signed-rank test. (**C** and **D**) Same as (A and B) but for putative DA neurons (type 1, *n* = 65; type 2, *n* = 63 neurons). (**E**) Number of neurons most activated in response to each cue among all type 1 (top, *n* = 81 in total) or type 2 (bottom, *n* = 78 in total) DA neurons. (**F**) Average responses of DA neurons aligned to onset of cues (presented 0 to 0.3 s) that were most activated by cue 1 among type 1 neurons (top, *n* = 58) or by cue 3 among type 2 neurons (bottom, *n* = 37). Mean ± SEM.

Identified type 1 DA neurons (*n* = 16) increased their spiking activity in response to 50R (time window: 0 to 0.5 s) and decreased activity in response to 50N (time window: 0.2 to 0.7 s) (Fig. 3, A and B). The activity was higher in response to 50R than expected reward after cue 1 (100R) and lower in response to 50N than expected NR (0N) (Fig. 3B). These responses were consistent with a bidirectional error signal for reward value, i.e., RPE signal, critical for moment-by-moment computation of changes in reward value and value-based learning (*8*–*12*, *15*, *16*). By contrast, we found that identified type 2 DA neurons (*n* = 15) showed inverse responses compared to the RPE neurons. Type 2 neurons were activated in response to 50N (time window: 0.3 to 0.8 s) and inhibited in response to 50R (time window: 0.4 to 0.9 s) (Fig. 3, A and B; see fig. S3A for further example type 2 neurons and fig. S3B for baseline subtracted average spiking rates). Furthermore, the activity was higher in response to 50N than expected NR (0N) and tend to be lower in response to 50R than expected reward (100R) (Fig. 3B). These two types of neurons were also evident in putative DA neurons (type 1, *n* = 65; type 2, *n* = 63) (Fig. 3, C and D, and fig. S3C). Thus, we hereafter show data using all recorded DA neurons including putative DA neurons. What about responses to the cues that predict reward (and NR) probabilities? Responses of the type 1 neurons after cue presentation appeared to be positively modulated by reward probability, consistent with a signal for expected reward value (Figs. 2B and 3, E and F; see fig. S3D for outcome periods) (*9*). By contrast, responses of the type 2 neurons were most likely to be activated by cue 3 that predict 0% reward (47.4% of type 2 neurons; Figs. 2D and 3, E and F; and fig. S3D; see fig. S3, E and F, for responses of negatively or positively modulated type 1 or type 2 neurons, respectively).

**Fig. 4.**
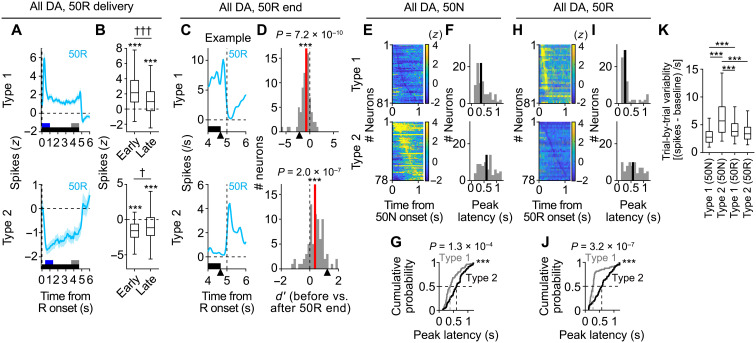
Type 2 DA error responses are slower than RPE-type DA responses. (**A**) Average response to 50R delivery (top, type 1, *n* = 81; bottom, type 2, *n* = 78). Means ± SEM. Black bar, duration of reward delivery. Blue and gray bars, time window to analyze activity in (B) (see Materials and Methods for detail). (**B**) Average activity of type 1 (top) or type 2 (bottom) neurons during the early or late responses to 50R delivery. ***P < 0.001 (versus baseline), †*P* < 0.05, and †††*P* < 0.001, two-sided Wilcoxon signed-rank test. (**C**) Average response of an example type 1 (top) or type 2 (bottom) neuron around the reward end. Arrowhead, the last reward shot. (**D**) Histogram of *d*′ comparisons of the activity of type 1 (top) or type 2 (bottom) neurons before versus after 50R termination. Red line, median. Arrowhead, *d*′ of each example neuron as in (C). Significant shift from zero, type 1: ***P = 7.2 × 10^−10^; type 2: ***P = 2.0 × 10^−7^, two-sided Wilcoxon signed-rank test. (**E**) Spiking activity of each type 1 neuron (top, *n* = 81) or each type 2 neuron (bottom, *n* = 78) to 50N. (**F**) Histograms of the peak latencies of the neurons [as in (E)]. Black line, median. (**G**) Cumulative probability of the peak latencies of type 1 versus type 2 responses to 50N [as in (E and F)]. ***P = 1.3 × 10^−4^, Kolmogorov-Smirnov test. (**H** to **J**) Same as (E to G) but for responses to 50R (type 1, *n* = 81; type 2, *n* = 78). ***P = 3.2 × 10^−7^, Kolmogorov-Smirnov test. (**K**) Trial-by-trial variability of spiking activity [as in (E and H)]. ***P < 0.001, two-sided Mann-Whitney *U* test with Bonferroni correction.

Next, we examined whether the activity of type 1 and type 2 neurons would also exhibit opposite responses to reward delivery. For reward in a trial, a total of 16 shots of 5 μl of saccharin water were dispensed every 0.31 s. A strong positive RPE should be induced at the onset of the reward delivery and should gradually decrease toward the termination. Furthermore, a negative RPE should be evident just after the termination because of the relative reward decrease. Consistent with these predictions, spiking activity of type 1 neurons showed all of these features (Fig. 4, A to D, for 50R; see fig. S4, A to D, for 100R). By contrast, type 2 neurons showed the opposite responses (Fig. 4, A to D, for 50R; see fig. S4, A to D, for 100R). Furthermore, at the population level, both peak latencies of the response of type 2 neurons after 50N (Fig. 4, E to G) and 50R (Fig. 4, H to J) were slower than those of the type 1 response (see fig. S4, E to G, for 100R). This was also true for responses after reward termination (fig. S4, H to M). These results show that the activity of type 2 neurons is slower than that of type 1 neurons in response to unexpected NR (50N) and unexpected reward (50R). Trial-by-trial variability of type 2 responses to 50N was much larger than that of type 1 responses (Fig. 4K).

**Fig. 5.**
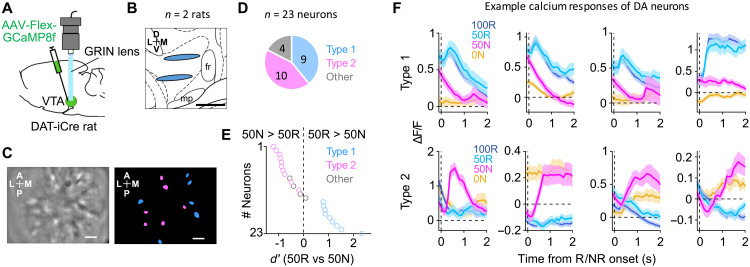
Type 2 DA error responses in calcium imaging. (**A**) Schematic of calcium imaging from DA neurons. (**B**) Locations of the tip of the GRIN lenses (blue ovals) in two rats shown on top of the atlas image. Scale bar, 500 μm. (**C**) Field of view of a DAT-iCre rat expressing GCaMP8f in the VTA (left) and locations of type 1 (blue) or type 2 (magenta) neurons (right). Scale bar, 100 μm. (**D**) Number of type 1, type 2, or other neurons among all recorded DA neurons (*n* = 23 neurons from two rats). (**E**) Distribution of *d*′ comparisons of activity in response to 50R versus 50N of all recorded DA neurons. (**F**) Example calcium responses of four type 1 (top) and type 2 (bottom) DA neurons during the outcome periods. Means ± SEM.

To confirm the activity of type 2 neurons in a different method, we used single-cell level calcium imaging. We expressed the calcium indicator GCaMP in DA neurons by injecting a Cre-dependent GCaMP virus into the VTA of DAT-iCre rats. We then implanted a gradient index (GRIN) lens above the VTA (Fig. 5, A and B). We found type 2 DA neurons in rats performing the operant task (*n* = 10 neurons of 23 neurons recorded from two rats) (Fig. 5, C to F). Thus, we confirmed type 2 DA neurons by recording spiking activity and intracellular calcium level. Together with the fact that rats appear to learn the task structure across trials (i.e., if they continue to work, about 50% reward can be expected) (Fig. 1, C to E), we hypothesize that the increased responses of type 2 neurons after 50N, reward termination, and cue 3 may signal error to actively (rather than passively) process the lack of reward based on the reward expectation and adjust the reward pursuit beyond the lack of reward. The decreased responses of type 2 neurons after reward appear to be consistent with this interpretation (i.e., a negative error).

### Type 2 DA neurons primarily signal error to actively cope with reward omission and provide a mechanism to switch toward future reward

**Fig. 6.**
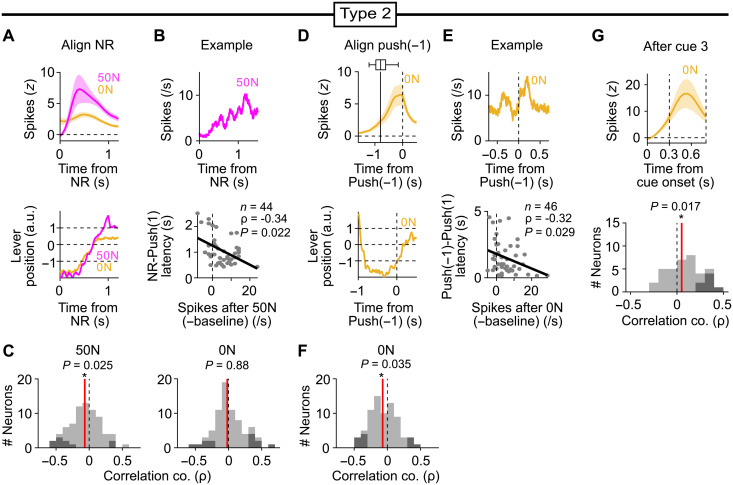
Type 2 DA neurons primarily signal error to actively process reward omission and provide a mechanism to switch toward future reward. (**A**) Average spiking activity of type 2 neurons (top, mean ± SEM across neurons, *n* = 73 neurons) and an example lever trajectory in a trial (bottom), aligned to NR (NR) onset after 50N or 0N. (**B**) Average spiking activity of an example type 2 neuron aligned to NR onset after 50N (top) and trial-by-trial correlation between the spiking activities of the example neuron (as in top) and NR-Push(1) latencies in the session (bottom, *n* = 44 trials, ρ = −0.34 and *P* = 0.022, Spearman’s rank correlation test). (**C**) Left: Histogram of the correlation coefficients (ρ) between the spiking activities after 50N and NR-Push(1) latencies [as in (B), bottom, for an example neuron] across type 2 neurons (*n* = 73). Red line, median. Black box, neurons with significant correlation coefficient (*P* < 0.05). Gray box, not significant. Significant shift from zero, *P = 0.025, two-sided Wilcoxon signed-rank test. Right: Same as left but for activity after 0N. *P* = 0.88. (**D**) Same as in (A) but those aligned to the time crossing the lever-position(−1) after 0N. Shown above is a box plot for average cue offsets in the recording sessions (top). Vertical line, median. (**E**) Same as in (B) but for an example activity aligned to time crossing the lever-position(−1) (top) and trial-by-trial correlation between the activity and Push(−1)-Push(1) latencies (bottom, *n* = 46 trials, ρ = −0.32 and *P* = 0.029). (**F**) Same as (C) but the correlation coefficients between the spiking activities after 0N and Push(−1)-Push(1) latencies. Significant shift from zero, *P = 0.035. (**G**) Average responses of type 2 neurons aligned to cue 3 onset that were most activated by cue 3 (top, *n* = 37). Mean ± SEM. Histograms of trial-by-trial correlation coefficients between each neuron’s activity and Cue-Pull latency after cue 3 (bottom). *P = 0.017.

If the type 2 neurons signal error to actively process reward omission and adjust the reward pursuit beyond the NR, then the spiking activity of the type 2 neurons after 50N (Figs. 3, A and C, and 4K) might relate to subsequent switching behavior (i.e., lever-pushing) to attempt to initiate the next trial. To examine this possibility, we first calculated, for each type 2 neuron, trial-by-trial correlation between spiking activity after 50N and the latency from the onset of NR to push the lever forward [“NR-Push(1)” latency] (Fig. 6, A and B). We then examined the distribution of the correlation coefficients across all type 2 neurons (Fig. 6C). We focused on the trials in which the rats responded relatively quickly and continued to engage (80.5% of all 50N trials; see Materials and Methods and table S1 for the criteria to select the trials). Significant negative correlation was observed in some of type 2 neurons [black boxes less than zero in Fig. 6C (left)]. Thus, the higher the spiking response of some type 2 neurons after unexpected NR (50N), the shorter the push latency. The correlation coefficients were overall negatively biased at the population level (*P* = 0.025; Fig. 6C). These results are consistent with the interpretation that some type 2 neurons may provide a mechanism to support trial-by-trial switching toward the next trial triggered by an unexpected NR. What about after rats pulled the lever following cue 3, that is, when NR was expected (i.e., expected NR: 0N)? We found no bias in correlation coefficients [*P* = 0.88; Fig. 6C (right), using 74.9% of all 0N trials, and table S1]. However, because rats would have already predicted NR before pulling back the lever, the timing of switching to attempt to initiate the next trial may be time-locked to the initiation of lever-pushing rather than NR onset. The negative correlation after 0N was evident in some type 2 neurons when the spiking activity was aligned to the initiation of lever-pushing (Fig. 6, D to F, and table S1; black boxes less than zero in Fig. 6F). The population-level correlation was negative (*P* = 0.035; Fig. 6F). Notably, this correlation was not apparent after 50N (fig. S5, A and B). This result suggests that the correlation between activity of some type 2 neurons after 50N and NR-Push(1) latency (as in Fig. 6, A and B) is not related to action initiation (*17*, *20*, *21*, *25*, *32*) independent of the NR onset but rather related to switching toward the next trial triggered by unexpected NR.

Furthermore, because the rats also pushed the lever toward the next reward after the end of reward delivery (Fig. 1, A and E), we examined the distribution of trial-by-trial correlations between spiking activity after the end of reward (50R) delivery and the latency of subsequent lever-pushing (fig. S5, C and D). Note that we observed increased responses of type 2 neurons just after the end of 50R delivery (Fig. 4, C and D), consistent with a positive response to the reward end (i.e., due to relative reward decrease). The population-level correlation was negative (fig. S5, C and D, and table S1; see fig. S5, E and F, for responses after 100R end). These results suggest that the increased activity of some type 2 neurons just after reward end may also relate to the switching to the pursuit of the next reward. However, the activity at reward end was much weaker than activity after 50N or 0N [see Fig. 6, A (top) and D (top) versus fig. S5, C and E]. By contrast, these correlations were much weaker in type 1 neurons (fig. S6, A to H). Moreover, the response to cue 3 of the type 2 neurons, but not the type 1 neurons, was positively correlated to subsequent latency to pull the lever (“Cue-Pull latency”) (Fig. 6G and fig. S6, I and J). Thus, the higher the response to cue 3, the longer the Cue-Pull latencies. The result suggests that the response to cue 3 of some type 2 neurons may relate to learning and behavior for switching toward the next trial away from reward pursuit in the current trial.

These results, together with much more distinct responses to the differences in expected outcomes (Fig. 2 to Fig. 5), suggest that the most parsimonious interpretation of the activity of type 2 DA neurons is that it primarily signals error to actively process reward omission, rather than the vigor or decision for a particular action, providing a mechanism for adjusting behavior toward future reward away from current reward.

### Type 2 DA error signal reflected in the NAc provides a mechanism for learning to efficiently overcome unexpected lack of reward

**Fig. 7.**
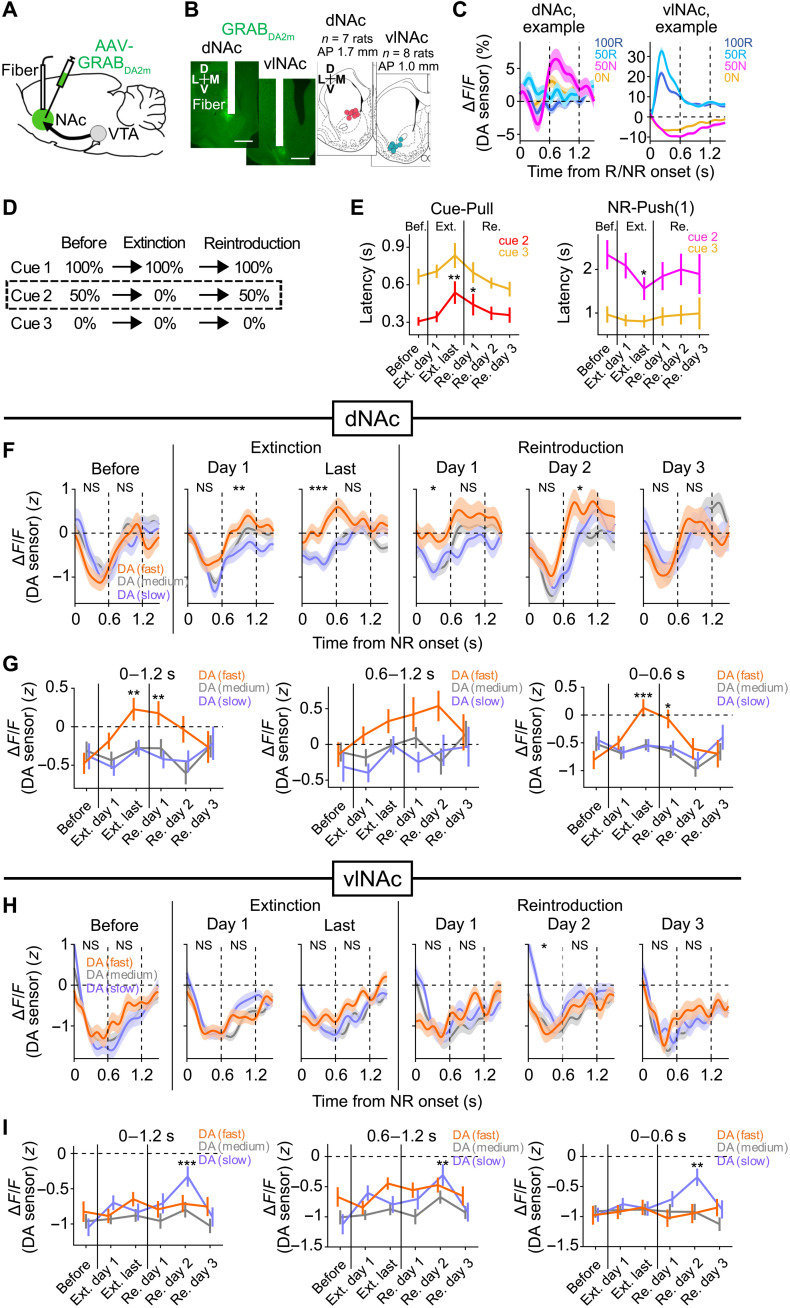
Type 2 DA error signal reflected in the NAc provides a mechanism for learning to efficiently overcome unexpected NR. (**A**) Measurement of DA levels in NAc using fiber photometry. (**B**) Left: Example expression of GRAB_DA2m_ (green) and optic fiber (white rectangle) in dNAc and vlNAc. Scale bar, 1 mm. Right: Fiber tip location in dNAc (left, *n* = 7, AP: +1.7 mm) and vlNAc (right, *n* = 8, AP: +1.0 mm). (**C**) Example average DA response recorded in a relatively late session recorded in dNAc (left) or vlNAc (right). Means ± SEM across trials. (**D**) Schematic of the 50R extinction and reintroduction task. (**E**) Cue-Pull (left) and NR-Push(1) (right) latency after cue 2 or cue 3 across the task. Means ± SEM. Before, 1 day before the 50R extinction. “Ext. last,” the last day of the extinction. “Re. day1,” day 1 of reintroduction of 50R. Significant difference of latency as compared to that before extinction. *P < 0.05 and **P < 0.01, two-sided Wilcoxon signed-rank test with Bonferroni correction, *n* = 11 rats. (**F** and **G**) Average DA responses after NR following cue 2 in dNAc with fast (25% of all trials, orange), medium (50%, gray), or slow (25%, purple) NR-Push(1) latencies across the task. Changes of DA levels aligned to NR onset (F) and average DA levels during 0 to 1.2 s (left), 0.6 to 1.2 s (middle), or 0 to 0.6 s (right) in each session (G). Significant effect of NR-Push(1) latency, *P < 0.05, **P < 0.01, and *** *P* < 0.001, Kruskal-Wallis test. NS, not significant (F). Significant difference of DA levels as compared to that before extinction. *P < 0.05, **P < 0.01, and *** *P* < 0.001, two-sided Wilcoxon signed-rank test with Bonferroni correction (G). (**H** and **I**) Same as (F and G) but for DA levels in vlNAc.

We next hypothesized that, if the type 2 DA neurons signal error to actively process reward omission, the signal might change in situations where new learning and behavioral adjustment to cope with reward omission is necessary. To examine this possibility, we decided to measure DA levels in the NAc, which are implicated in learning and motivated behaviors (*24*, *33*–*38*). To monitor DA release using fiber photometry, we expressed a genetically encoded DA sensor GRAB_DA2m_ (*39*) in the dorsal part of anterior NAc (dNAc). We confirmed that dNAc receives anatomical inputs from DA neurons in anterior lateral VTA (Fig. 7, A and B, and fig. S7A), where we found that about half of recorded DA neurons were type 2 (Figs. 1G, 2E and 5, B and D). We also targeted the ventrolateral part of NAc (vlNAc), the region that is likely to receive inputs mainly from RPE neurons (Fig. 7B) (*18*, *33*) .

We first trained rats in the same operant cue-reward association task (as in Fig. 1A), except that we used auditory instead of odor cues. In relatively late sessions, DA levels in dNAc after unexpected NR (50N) somewhat decreased initially [until about 0.4 s in case of the response in Fig. 7C (left)], similar to the decrease of RPE signal in vlNAc (Fig. 7C, right) but rapidly increased afterward (Fig. 7C, left). The overall temporal dynamics of the DA level in dNAc was consistent with a mixed signal (Fig. 7C, left), receiving sequential inputs from the signal from type 1 (RPE) neurons and the signal from type 2 neurons (see Fig. 4, E to J). This is consistent with the observation that the two types of DA neurons are spatially mixed in anterior lateral VTA (Fig. 5C). By contrast, DA levels in vlNAc continued to decrease, consistent with negative RPE signal (Fig. 7C, right) (*18*) . These results suggest that DA levels in dNAc reflect relatively stronger type 2 DA signal than those in vlNAc (see also fig. S7, B to D, for relatively early sessions and 405-nm control).

We next addressed whether the population-level correlation between activity of type 2 DA neurons and NR-Push(1) latency (Fig. 6, A to C) might be reflected in the DA levels in dNAc. Although negative correlations between the DA levels in dNAc at 0.6 to 1.2 s after 50N and NR-Push(1) latency were found in some early sessions (fig. S7, E and F), they were not evident in relatively late sessions in which the behavior was stable. These results suggest that the DA levels in dNAc are unlikely to primarily reflect the vigor or decision of the lever-pushing behavior. Rather, we assumed that the DA levels reflect error to actively overcome reward omission.

We reasoned that the type 2 signal in dNAc after NR following cue 2 may become stronger in tasks that induce stronger type 2 error, such as tasks that require new learning and behavioral adjustments to actively cope with the NR. To test the possibility, we first omitted reward after cue 2 (“Extinction”) for 3 to 6 days [4.9 ± 0.4 (mean ± SEM) days per rat] after the cue-reward association task (Fig. 7D). Even during the extinction of the reward after cue 2, rats still had a chance to obtain reward after cue 1 (i.e., 33% chance to obtain reward across all trials). Therefore, an efficient behavioral strategy to ultimately obtain more reward in the task is to stop engaging in potential reward after cue 2, similar in the response to cue 3, and switch earlier toward the next opportunity to obtain reward. We assumed that in response to the NR after cue 2, the rats would initially learn that the percentage of NR increased. We also assumed that as rats were exposed to more extinction trials, they would gradually show slower lever-pulling response and switch to the next reward at an earlier time. Cue-Pull latency and NR-Push(1) latency after cue 2 in the extinction day 1 were almost the same as before the extinction, but the latencies in the last day of the extinction became longer and shorter, respectively (Fig. 7E). What about DA transients along with this behavior? In the extinction day 1, we found a much stronger negative correlation between DA level in dNAc during 0.6 to 1.2 s and NR-Push(1) latency than that before extinction (Fig. 7, F and G). This change of the DA levels is consistent with the type 2 error signal for learning increased opportunity of the lack of expected reward to adjust the reward pursuit beyond that lack of reward, which is likely to be more positive as rats attempt to more actively process the lack of reward [i.e., faster NR-Push(1) latency]. By contrast, in the last extinction day, the timing of the negative correlation between DA transients in dNAc and NR-Push(1) latency was earlier (Fig. 7, F and G). This is consistent with the interpretation that the type 2 error signal provides a mechanism for learning to switch to proceed to the next reward at an earlier time, an efficient behavioral adjustment to ultimately obtain more reward.

Furthermore, we examined how the correlation might change when the 50% reward after cue 2 was introduced again (“Reintroduction” in Fig. 7D). Here, the rats would learn that the percentage of reward increased and gradually switch to the next reward at a later time. The change of Cue-Pull latency and NR-Push(1) latency were consistent with this interpretation (Fig. 7E). Accordingly, the timing of the negative correlation between DA levels and NR-Push(1) latency became slower over the first 2 days, and the correlation was no longer observed on the day 3 (Fig. 7, F and G). Given that average NR-Push(1) latencies after cue 2 were not different across the 3 days (Fig. 7E and fig. S7G), the change of the negative correlation is again unlikely to reflect the vigor or decision of the lever-pushing behavior. Rather, the correlation is consistent with a positive error signal for learning to adjust behavior in response to the increased reward expectation (i.e., a relatively larger error in response to NR where the reward expectation increases compared to NR where NR is highly likely). These changes of the correlation were not evident for RPE-type DA transients in vlNAc (Fig. 7, H and I). Together with the activity of two types of DA neurons in the VTA (Figs. 2 to 6), these results suggest that type 2 DA responses reflected in dNAc much more strongly signal error to actively cope with reward omission than type 1 (RPE-type) DA responses reflected in vlNAc.

### Type 2 DA error signals are initially evident but become weaker in the transition from the operant task to a Pavlovian task where reward outcomes can be passively processed

Last, we addressed whether the type 2 DA error signal could be also observed in typical tasks that have been commonly used to examine midbrain DA activity, i.e., a Pavlovian task. Although the RPE-type DA signal has been reported in Pavlovian tasks many times (*8*, *9*, *14*, *16*, *18*), type 2 DA error signals in the tasks are unknown. In typical Pavlovian tasks, animals may not necessarily need to actively process reward omission. Instead, even if animals might initially attempt to obtain reward just after reward omission, they are unable to do so due to the nature of the task. Thus, they typically passively process reward omission and just wait for the next reward to be presented. We hypothesized that if we switched the task from the operant task to a Pavlovian task while keeping the cue-reward relationship similar, the signal for overcoming NR might be apparent initially but gradually become weaker.

**Fig. 8.**
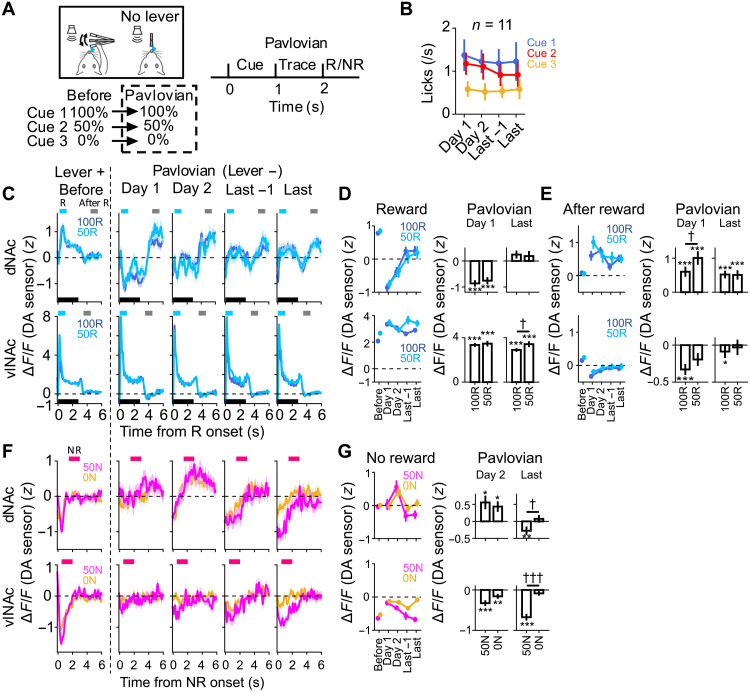
Type 2 DA error signals are prominent initially but become weaker in the transition from the operant task to a Pavlovian task where reward outcomes can be passively processed. (**A**) Left: Transition from the operant task to the Pavlovian task. Right: Structure of a trial. ITI was 35 s on average. (**B**) Number of licks during the cue and trace period in the task. Means ± SEM. Last or “Last −1” indicates last or the day before the last day in the Pavlovian task. (**C**) Average DA responses to reward in dNAc (top) or vlNAc (bottom) in the task. Means ± SEM across trials. *n* = 7 (top) or 8 (bottom) rats. See Materials and Methods for the total number of trials. Black bar, duration of reward (2.8 s). Light blue or gray bar, time window used for analysis in (D) or (E), respectively. (**D**) Left: Average DA levels in dNAc (top) or vlNAc (bottom) across all trials in response to 100R or 50R. Means ± SEM. Right: DA levels on day 1 or the last day. Means ± SEM. See fig. S8A for box plots. *P < 0.05, **P < 0.01, and ***P < 0.001, two-sided Wilcoxon signed-rank test; †*P* < 0.05 and †††*P* < 0.001, two-sided Mann-Whitney *U* test. (**E**) Same as (D) but for response after reward. (**F**) Average DA responses to NR in dNAc (top) or vlNAc (bottom). Means ± SEM. Magenta bar, time window of a response to NR as in (G). (**G**) Same as in (D) but for response to 50N or 100N.

To test this hypothesis, after training rats in the operant task (Fig. 7), we removed the spout lever and replaced with a spout fixed in front of the mouth (Fig. 8A, “Pavlovian”). Across the task transition, the relationship between cue and reward probability and the reward amount was kept the same. One of the three cues was presented for 1 s, and then, the reward was delivered starting from 1 s after the end of a cue, followed by intertrial interval (ITI) for about 35 s (Fig. 8A). Although the rats had never been trained with the temporal relationship between the cue and reward presentations, they quickly adapted to the new task condition and exhibited anticipatory licking, which depended on reward probabilities throughout all Pavlovian sessions (from four to six sessions per rat) (Fig. 8B).

**Fig. 9.**
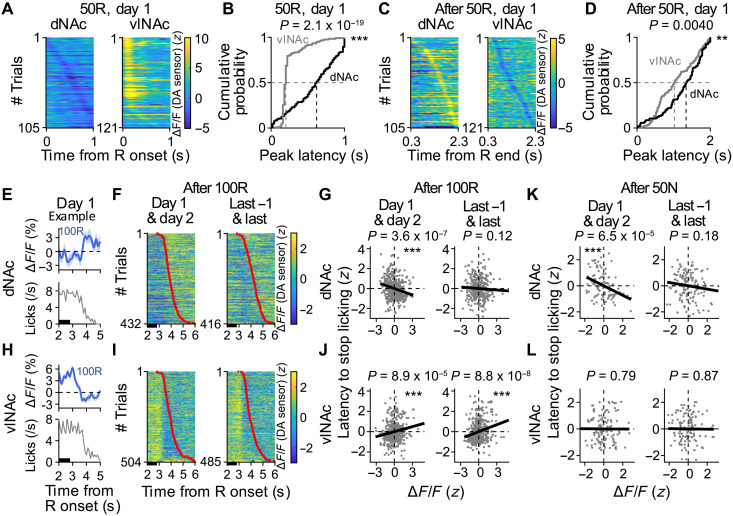
Type 2 DA responses provide a mechanism for learning to adjust behavior toward the pursuit of the next reward early in the Pavlovian task. (**A**) DA transients in dNAc (left, *n* = 105 trials across seven rats) or vlNAc (right, *n* = 121 trials across eight rats) in response to 50R on day 1. (**B**) Cumulative probability of the peak latencies in response to 50R [as in (A)]. ***P = 2.1 × 10^−19^, Kolmogorov-Smirnov test. (**C** and **D**) Same as (A and B), but for responses after 50R termination. **P = 0.0040 (D). (**E**) Example average DA response in dNAc (top) and number of licks (bottom) after 100R end in the day 1. Black bar, reward delivery (the last shot at 2.8 s). (**F**) DA transients in dNAc in the first 2 days (left, *n* = 432 trials across seven rats) and in the last 2 days (right, *n* = 416 trials). (**G**) Trial-by-trial correlation between latency to stop licking and DA level in dNAc in the first 2 days (left, *n* = 432 trials, ρ = −0.24 and ***P = 3.6 × 10^−7^, Spearman’s rank correlation test) or the last 2 days (right, *n* = 416 trials, ρ = −0.076, and *P* = 0.12). (**H** to **J**) Same as (E to G), but for vlNAc. *n* = 504 trials, ρ = 0.17, and ***P = 8.9 × 10^−5^ [(J), left]; *n* = 485 trials, ρ = 0.24, and ***P = 8.8 × 10^−8^ [(J), right]. (**K** and **L**) Same as (G and J) but for DA levels in response to 50N. *n* = 110 trials, ρ = −0.37, and ***P = 6.5 × 10^−5^ [(K), left]; *n* = 139 trials, ρ = −0.12, and *P* = 0.18 [(K), right]; *n* = 134 trials, ρ = −0.023, and *P* = 0.79, [(L), left]; *n* = 129 trials, ρ = 0.014 and *P* = 0.87 [(L), right].

We found that DA responses in dNAc changed markedly with the task transition (Fig. 8, C to G, and fig. S8, A to C). First, DA levels in dNAc decreased at reward onset (time window, 0.2 to 1.2 s after reward onset) on days 1 and 2 (Fig. 8, C and D, and fig. S8A). This response is consistent with a type 2 negative error signal for active processing of reward omission. Note that this negative response was also evident in spiking activity and calcium response of type 2 DA neurons in the operant task (Figs. 2 to 5). The DA response in dNAc became less evident over the course of learning (Fig. 8D and fig. S8A). By contrast, RPE-type DA increases in vlNAc at reward onset was apparent throughout the learning (Fig. 8D and fig. S8A). Second, DA levels in dNAc increased just after the termination of the reward delivery (time window, 4.5 to 5.5 s after reward onset) (Fig. 8, C and E, and fig. S8B). This is consistent with a type 2 positive error signal for reward omission due to the relative reward decrease, as was evident in type 2 DA neurons (Fig. 4, C and D). This increase was most prominent on day 1 and became less evident through learning but was still above baseline in the last day (Fig. 8E and fig. S8B). The similar change was observed for RPE-type decrease of DA levels in vlNAc (Fig. 8, C and E, and fig. S8B). Both type 2 DA responses to reward onset and reward end in dNAc were slower than those of RPE-type DA responses [Fig. 9, A to D, and fig. S8, D to G (for 100R)], consistent with the results of spiking activity of DA neurons (Fig. 4, E to J, and fig. S4, E to M). Third, DA levels in dNAc increased after NR on day 2 (Fig. 8, F and G, and fig. S8C), consistent with a positive surprise signal for reward omission as was evident in type 2 DA neurons (Figs. 2 to 6). Note that a positive surprise signal for reward omission would be induced only after some expectation of reward is established, which may be the reason why the signal was less clear on day 1 than day 2. The increase was not observed in the last sessions (Fig. 8, F and G, and fig. S8C). By contrast, RPE DA decreases in vlNAc after unexpected NR (50N) became evident through the learning (Fig. 8, F and G, and fig. S8C). Given that the RPE signal in vlNAc was relatively stable compared with that in dNAc, the initial changes in the DA signal in dNAc (at the reward onset, reward end, and 50N) are likely to primarily reflect changes in the activity of type 2 DA neurons.

Could the DA transients in dNAc also provide a mechanism underlying behavioral adjustment toward the pursuit of the next reward away from the current reward? To address this question, we examined the relationship between DA levels and behavior to retrieve current reward, i.e., licks. If the DA increases in dNAc just after the reward end might relate to the switch away from the current reward seeking, then the DA levels should negatively correlate to latency to stop licking (i.e., the more DA in dNAc, the faster the rats stop licking). DA levels in dNAc after 100R negatively correlated to the latency in the early sessions (Fig. 9, E to G, and fig. S8J; see Materials and Methods for the definition of the latency to stop licking). However, this correlation was not observed in the last sessions (Fig. 9, F and G, and fig. S8J). The same results were obtained when we used number of licks instead of latency to stop licking (fig. S8, H and K). By contrast, DA levels in vlNAc positively correlated to the latency (Fig. 9, H to J, and fig. S8J; see fig. S8, I and K, for number of licks). Moreover, this negative correlation between the DA levels after unexpected NR (50N) and latency to stop licking was also evident in dNAc early in learning (days 1 and 2) but not vlNAc (Fig. 9, K and L).

Collectively, these results show that the type 2 DA responses in dNAc, which provide a mechanism for learning to adjust behavior toward the pursuit of the next reward, can be evident early in the transition to the Pavlovian task. However, the signal seemed not to be strongly induced later in the task. This may be because once the rats learned the task structure, they may not need to continue to actively cope with NR. Rather, they could just passively process it. Thus, these results further support the interpretation that the type 2 DA responses signal error to actively, rather than passively, cope with lack of expected reward.

### Causal relevance of type 2 DA error signal in dNAc and behavioral adjustment to actively cope with lack of expected reward

Is the type 2 DA error signal transmitted to dNAc causally related to behavioral adjustment to cope with reward omission? Type 1 and type 2 neurons are spatially mixed in anterior VTA (Figs. 1G and 5, B and C), and DA levels in dNAc reflect both inputs (Fig. 7), despite relatively stronger inputs from type 2 neurons especially when rats appeared to actively process reward omission (Figs. 7F and 8, C to G). Thus, directly testing the causality of type 2 DA signal in dNAc is technically challenging because it requires selective manipulation of the activity of type 2 DA neurons in a deep brain area that project to dNAc in a task- and timing-specific manner. Nevertheless, to address the causality, we optogenetically stimulated the dopaminergic input into dNAc during reward omission in a reward extinction task where rats needed to actively cope with reward omission (fig. S9; see Supplementary Text for details). Rats with the optogenetic activation executed more trials in the early (but not late) extinction blocks than in rats without the activation. The behavioral effects suggest that the DA inputs to dNAc are causally related to active switching toward the next reward after NR as long as the next reward can be expected. This result suggests that type 2 activity is likely causally related to behavioral adjustment to cope with lack of expected reward, although we do not exclude the possibility that the effect might be, in part, due to activation of type 1 (RPE-type) DA inputs (*37*).

## DISCUSSION

Here, we developed operant tasks for rats that enabled us to quantitatively measure the ability to actively and adaptively switch toward the next opportunity to obtain a probabilistic reward after the lack of reward. Recording of spiking activity and single-cell calcium imaging from DA neurons in the anterior part of lateral VTA revealed that about half of recorded DA neurons (type 2 neurons) showed increased responses to unexpected reward omission and decreased responses to unexpected reward. The type 2 DA responses were consistently slower than the RPE-type DA responses. Measurements of the DA levels showed that the trial-by-trial correlation between the type 2 DA response in dNAc and behavioral switch toward the next reward became evident upon extinction and reintroduction of 50% reward that required new learning to adjust behavior to cope with unexpected NR actively and efficiently. Furthermore, the type 2 DA responses in dNAc were evident early in the transition from the operant task to the Pavlovian task, a task that did not require specific active actions for obtaining reward (e.g., manipulation of the spout lever), but the signal became weaker later in the task when the reward outcomes could be passively processed. These results demonstrate that the type 2 DA responses primarily signal error to actively cope with lack of reward based on the reward expectation. Our results show that the type 2 DA error signal provides a mechanism for adaptively pursuing uncertain reward to overcome the lack of reward and ultimately obtain more reward.

Although previous studies showed that neurons in the lateral habenula (LHb) exhibited similar bidirectional responses with those of the type 2 DA neurons (*40*, *41*), LHb is thought to rather facilitate RPE-type DA signal (*40*–*44*). Furthermore, some neurons in the central nucleus of the amygdala (*42*), paraventricular nucleus of the thalamus (*43*), and anterior cingulate cortex (*44*–*46*) are more activated in response to the lack of expected reward than reward receipt. However, their roles in actively processing the lack of expected reward and adjusting the pursuit of that reward are unclear. The behavior that we required rats in the present study appear to be different from typical behaviors in other tasks that allowed animals to explore multiple choice options to obtain reward after lack of expected reward (*11*, *24*, *47*, *48*). Animals may not repeat the same behavior if the expected reward is omitted many times and the expectation of the reward decreases. Rather, they would explore other options that would lead to a higher probability of reward [i.e., “lose-shift” strategy (*47*)]. Such operant tasks in which value-oriented behavior is dominant might not continuously induce predominant activity of type 2 DA neurons. However, we speculate that it may depend on the task demand. Thus, even in environments where multiple choice options are available, the types 2 DA error activity could contribute to regulating the so-called exploration (switch between options)–exploitation (remain at one option) balance, which aims to maximize obtained rewards, perhaps through interactions with cortices (*44*, *45*, *46*, *49*). Future studies are necessary to address this possibility.

Type 2 DA responses, which we found in the anterior part of lateral VTA, appear to be different from other DA responses activated by aversive stimuli (e.g., air-puff or electrical shock) (*18*, *22*, *23*) and reinforce avoidance of the aversive stimuli (*23*). Those aversion-responsive DA neurons were found in lateral substantia nigra pars compacta and ventromedial region of the NAc shell (*18*, *19*, *22*, *23*, *26*). Type 2 DA neurons are unique in that they are not only activated by reward omission but also inhibited by unexpected reward. Furthermore, type 2 DA responses are more prominent when behavioral adjustment to actively cope with lack of reward is required. We found that the type 2 responses were opposite to and slower than those of type 1 (RPE) neurons. For example, in response to 50N, peak latency of increased spiking activity of type 2 neurons was 0.22 s slower than that of decreased activity of type 1 neurons when compared using median (0.38 s versus 0.60 s) of the peak latencies. Furthermore, the type 2 responses were more variable in trial-by-trial responses (Fig. 4K) and peak latencies (Fig. 4, E to J), resulting in broader average responses than type 1 responses (Figs. 3 and 4). These differences suggest that the two types of DA responses in dNAc are unlikely to cancel out each other. Rather, it implicates sequential and complementary roles of the RPE-type DA responses in signaling error for reward value and type 2 DA responses in signaling error for active processing of the lack of reward. The two types of error signals would be sequentially transmitted to medium spiny neurons (MSNs) in dNAc and regulate learning and behavior through, in part, modulation of synaptic plasticity of different types of MSNs (*50*, *51*). Thus, by coordinating these opposite responses, the two types of DA signals would enable adaptive and robust pursuit of uncertain reward. In the operant cue-reward association task, the type 2 DA responses in dNAc tended to become clearer in relatively late sessions [e.g., session 30 (Fig. 7C); see responses in early sessions (recorded in session 14.3 on average; Figs. 7F and 8, C and F, and fig. S7D], consistent with receiving equivalent inputs from both type 1 and type 2 DA neurons (recorded in session 32.5 on average) (Fig. 3). Future research is needed to address how the extent of learning of the cue-reward relationship affects the relative strength of type 1 and type 2 DA responses.

Theoretical models assume that acquired salience (the ability of a stimulus to capture attention) assigned to an event associated with both reward and reward omission (i.e., probabilistic reward), such as the cue 2 in the present study, can be higher than salience assigned to an event associated with reward only, such as the cue 1 (*1*, *29*, *52*). The two types of DA error signals might provide a neural mechanism underlying potentially higher acquired salience to the cue 2 (and following events) than the cue 1, which might explain, at least in part, relatively fast Cue-Pull latency after cue 2 comparable to that after cue 1 (Fig. 1C) [see also (*1*)]. This is because both the type 2 DA increase in response to reward omission after the cue 2 and the type 1 DA increase in response to reward after the cue 2 can mainly contribute to increasing acquired salience to the cue 2 and following events, perhaps via MSNs in dNAc (*50*) and the prefrontal cortex (*1*), whereas only the type 1 DA increase in response to reward after cue 1 can mainly contribute to increasing acquired salience to the cue 1. Together, our results provide a foundation to substantially expand theoretical frameworks for the function of DA (*53*, *54*) and to advance our understanding of the computational algorithms in the brain that underlie learning and decision-making to pursue uncertain reward and ultimately obtain more reward.

Partial reinforcement of one behavior is known to be able to produce generalized persistence in other behaviors in both humans and animals (*2*, *5*, *55*). Thus, partial reinforcement could be a way that increases persistence in general (*5*) and may provide a basis for maximizing the number of goals that can be achieved in the future. We found that type 2 DA activity was commonly induced in both the operant and Pavlovian tasks. Thus, our findings open the door for further research into the neural mechanisms underlying the adaptive ability to pursue probabilistic rewards across a variety of behaviors over long time scales. Last, our findings of the type 2 DA neurons potentially provide insights into the neural mechanisms of psychiatric disorders in which DA is deeply involved [e.g., (*56*, *57*)]. For example, a fundamental feature of addiction is to continue to pursue a particular target despite negative consequences (*57*) [e.g., loss chasing in pathological gambling (*58*, *59*)]. RPE-type DA neurons may not be directly involved in the feature because they are inhibited by negative outcomes. Rather, an abnormal hyperactivity in type 2 DA neurons and/or an imbalance between RPE-type DA neurons and type 2 DA neurons might more directly contribute to the feature.

## MATERIALS AND METHODS

### Animals

Adult male and female rats, either wild-type Long-Evans (Charles River Laboratories, Oriental BioService) or heterozygous *DAT(Slc6a3)-iCre* (DAT-iCre), expressing improved Cre recombinase (iCre) under the control of the DA transporter promoter, on a Long-Evans background [LE-Tg (DAT-iCre) 6Ottc; RRRC, stock no. 758] (*31*) were 9 to 13 weeks old and between 260 and 500 g at the start of experiments. We used 24 DAT-iCre (19 males and 5 females) and 11 wild-type rats (11 males). One female rat was used for the calcium imaging experiment. Four female rats were used for the optogenetic experiment (fig. S9), and two each were for channelrhodopsin 2 (ChR2) or enhanced yellow fluorescent protein (eYFP) rats. Rats were maintained on a 12-hour light/12-hour dark cycle and tested during the light phase. Until the start of behavioral training, rats had access to food and water ad libitum. During behavioral training, rats had free access to food but were water-restricted while keeping their body weight more than 80% of the weight before the start of all behavioral experiments. All experimental procedures were approved by the Animal Care and Use Committee at Kyoto University and the National Institutes of Natural Sciences and performed in accordance with the approval.

### Recombinant viral vectors

The following adeno-associated viral vectors were used to express transgenes of interest in either Cre-recombinase–dependent or Cre-recombinase–independent manner: AAV_5_-EF1α-DIO-ChR2 (H134R)-eYFP [2.3 × 10^12^ genome copies/ml (GC/ml); University of Pennsylvania, Addgene, Section of Viral Vector Development, National institute for Physiological Sciences (NIPS)] (*60*), AAV_5_-EF1α-DIO-eYFP (8.3 × 10^11^ GC/ml; University of North Carolina), AAV_9_-hSyn-DA_2m_ (5.3 × 10^12^ GC/ml; Vigene Biosciences), AAV_5_-Syn-Flex-jGCaMP8f (4.0 × 10^12^ GC/ml; Section of Viral Vector Development at NIPS), and AAV_DJ_-Syn-Flex-GCaMP7f (4.5 × 10^11^ GC/ml; Section of Viral Vector Development at NIPS).

### Surgery

Specific details of subjects and surgery for each experiment are described below. For all behavioral experiments, rats underwent sterile stereotaxic surgery under isoflurane anesthesia to install a headplate (CFR-2, Narishige). The skull was exposed, cleaned, and hardened using super bond (Super-Bond C&B, Sun Medical). To provide a firm and solid base for the headplate, 14 stainless steel skull screws (SNZS-M1-2, NBK) were drilled into the skull and secured in position by a dual curing dental adhesive (Panavia F2.0, Kuraray Noritake Dental) before affixing the headplate to the skull with a self-curing dental acrylic resin (Unifast II, GC Corporation). All surgical procedures for fiber photometry and optogenetic experiments, such as headplate installation, virus injection, and fiber placement, were performed on the same day. Rats were allowed to recover for 1 week before training started.

### Operant cue-reward association task

Head-restrained rats were placed in a stainless-steel cylinder and trained to manipulate a “spout lever” with their right forelimb (TaskForcer, O’hara), a lever integrated with a spout to deliver liquid reward (*27*, *28*). Rats could obtain the reward from the tip of the lever (as illustrated in Fig. 1A). The range within which rats could pull or push the lever (i.e., the distance between the “pull” and “push” stoppers) was regulated by magnets and was approximately 13.2 mm. This range corresponded to lever-position(−2: arbitrary unit) (“pull”) to position(2) (“push”). Rats usually manipulated the lever in a range from position(−1.5), which was approximately the same as the mouth position, to position(2). The lever returned automatically toward the center position(0) if rats released it. The lever position was always monitored and recorded.

#### Odor task

Before the odor task, rats were trained to self-initiate a trial by pushing forward the lever to more than the lever-position(1) after illumination of a cue light. The cue light was kept on until reward outcomes were presented (see below for details). After rats kept the lever-pushing for 0.4 s, odor-free air was delivered for 0.3 s. Then, a high-tone sound (square 3 kHz, 65 dB, and 0.12 s) was presented (OPR-8210, O’hara). After the sound onset, rats were allowed to pull back the lever toward their mouth. Saccharin water reward (0.1%, 20 μl, 5 μl × 4 shots dispensed every 0.31 s) was delivered from the spout (OPR-7300, O’hara) with 100% probability, starting at 0.4 s after rats pulled the lever closer than the lever-position(−1). Cue-Pull latency was defined as the latency from odor cue offset to pull the lever closer than lever-position(−1). The distance from position(−1) to (1) was approximately 6.6 mm.

The basic design of a trial in the odor task (Fig. 1A) is the same except for the followings. After rats pushed forward the lever for 0.4 s, an odor cue was presented for 0.3 s. One of three different odors was delivered in each trial in a pseudorandom order, with a custom-designed olfactometer (Odor test unit, O’hara). Odors were selected pseudorandomly from 1-butanol, eugenol, (+)-limonene, 2-octanol, citral (B0944, A0232, L0047, O0037, D0762, respectively, Tokyo Chemical Industry), pentyl acetate (018-03623, Wako), and (R)-(−)-carvone (124931, Sigma-Aldrich) for each rat. Each odor was dissolved in mineral oil at 1:10 dilution. Each odor was associated with different probability of reward: 100, 50, or 0%. In a rewarded trial, the water reward of 80 μl (5 μl × 16 shots dispensed every 0.31 s) was delivered. The cue light was turned off 2 s after the last shot of reward delivery. In a nonrewarded trial, the cue light was turned off 2 s after rats pulled the lever closer than lever-position(−1) following 50N or closer than lever-position(1) following 0N. We referred to trials in which rats did not pull the lever closer than lever-position(−1) before cue light was turned off as “0% stop” trials (fig. S1D, 14.7% of all of 0N trials) and excluded the trials from all of the analysis of behavior and neural activity after 0N. After cue light was turned off, an ITI was introduced for 4 s. After ITI, the cue light was turned on after rats held the lever in the middle range [i.e., between lever-position(−1) and lever-position(1)] for 0.1 s, indicating the start of the next trial. Rats were required to pull the lever closer than the lever-position(−1) after cue 1 and cue 2 or closer than the lever-position(1) after cue 3 to proceed to the next trial. In error trials in which rats pulled the lever before the offset of odor cues, a high-tone sound (4 kHz, 65 dB, 0.5 s) was presented, and the cue light was turned off. In error trials in which rats did not pull the lever within 1.5 s, the cue light was turned off. After these error trials, ITI was 7 s, and the same odor was presented in the next trial. The data of the error trials were excluded from the analysis. Rats executed 358.6 ± 10.6 (mean ± SEM) (range 148 to 746) trials per recording session (*n =* 101 sessions, 7 rats) in approximately 2 to 3 hours.

#### Auditory task

The auditory task was introduced for single-cell calcium imaging and photometry experiments. The basic design of a trial in the auditory task was the same as that in the odor task, except for the following. After illumination of the cue light, rats could initiate a trial by pushing the lever for 0.7 s, which resulted in delivery of an auditory cue for 0.2 s. One of three auditory cues, selected pseudorandomly from 4, 6, 8, 10, square 3, and square 11 kHz for each rat, was delivered in each trial in a pseudorandom order (65 dB, OPR-8210, O’hara). Cue-Pull latency was defined as the latency from auditory cue onset to pull the lever closer than lever-position(−1). In a rewarded trial, 0.1% saccharin water (50 μl, 5 μl × 10 shots dispensed every 0.31 s) was delivered. In a nonrewarded trial, the cue light was turned off 2.4 s after rats pulled the lever. Cue light continued to be on if rats pulled the lever before the offset of auditory cues (i.e., no error). ITI was 4.4 s.

#### 50% reward extinction task

The basic design of a trial was the same as the sound task, except that reward was omitted (i.e., “0% reward”) after cue 2 that was previously associated with 50% reward. This extinction continued for three to six sessions (average 4.9 sessions per rat).

#### Pavlovian conditioning following the sound task

In the Pavlovian conditioning (Figs. 8 and 9), reward was presented from a spout in front of head-restrained rats, instead of the spout lever. Before the conditioning, rats were habituated in the setting approximately 15 min per day for 3 days; 0.1% saccharin water was manually given via the spout several times per day to ensure that rats retrieve reward from the spout immediately after the presentation. Once the conditioning started, the relationships between cues and reward probabilities were the same as those used for the sound task. One of the three auditory cues was presented for 1 s, followed by a delay of 1 s and an outcome (reward or NR). In a rewarded trial, 0.1% saccharin water (50 μl, 5 μl × 10 shots dispensed every 0.31 s) was delivered. ITI was 35 s on average (selected pseudorandomly from 15 to 55 s). This conditioning continued for four to six sessions (average 5.2 sessions per rat). Licks were measured by infrared photo beam sensor (Licking unit, O’Hara).

### In vivo extracellular recording of electrical activity of DA neurons

We used a custom-made single-drive movable optrode, consisting of four tetrodes (constructed from 12.5-μm tungsten wire) (California Fine Wire) mounted in a 33-gauge stainless-steel cannula (Small Parts) (*30*), with an optic fiber (200-μm core) attached to a 2.5-mm metal ferrule (MFC_200/230-38mm_MF2.5_FLT, Doric) placed in the center of the cannula. The ends of the tetrodes were extended up to 300 to 500 μm below the fiber tip. We first injected 0.75 μl each of AAV_5_-EF1α-DIO-ChR2(H134R)-eYFP into the left VTA (AP: −5.2 mm, ML: 1.5 mm, DV: +7.7 mm, at an angle of 5°; AP: −5.7 mm, ML: 1.4 mm, DV: +8.4 mm, at an angle of 5°) and then implanted the optrode (AP: −5.2 mm, ML: 1.5 mm, DV: +7.3 mm, at an angle of 5°). The recording locations ranged from AP: −4.8 to −5.6 mm (see Fig. 1G in which the locations were projected onto the slice of AP: −5.2 mm) (*61*). During the recording sessions, wideband neural signals were acquired continuously at 20 kHz on a 256-channel Amplipex systems (KJE-1001, Amplipex). Optrodes were lowered at least 40 μm at the end of each recording session. To extract spikes, the neural signals were filtered at 600 to 9000 Hz. Spike sorting was performed semiautomatically, using MClust-4.3 (A.D. Redish), followed by manual adjustment of the clusters on the basis of waveform characteristics, spike autocorrelation, and clustering.

To identify DA neurons, we used ChR2 to trigger spikes with blue light (*16*, *17*, *24*). At the end of each recording session, we delivered trains of 10 462-nm light pulses, each 5 ms long, at 5, 10, and 20 Hz, with an intensity of approximately 10 mW/mm^2^ at the tip of the fiber. Neural activity aligned to light pulse onset was down-sampled to 10 kHz, averaged across trials (bin: 0.1 ms), and smoothed by 1-ms moving average to construct a PETH. A significant increase was defined as at least 12 consecutive bins (total, 1.2 ms) having a spiking rate larger than a threshold of 5 SDs above baseline activity defined as the activity from −3 to −2 s before light onset. We also compared the light-evoked waveforms (evoked within 10 ms after light onset) to average waveforms recorded during the task, confirming that all light-evoked units had a Pearson correlation coefficient of >0.9. DA neurons were identified in seven rats (8, 8, 6, 5, 4, 4, or 1 units in each rat). Peak width of waveforms was defined as the full width at half maximum of the largest negative or positive component of the averaged spike waveform. Nontagged neurons with spiking rate more than 20 Hz and peak width less than 200 μs were classified as non-DA cells (*24*). The recording was conducted at 32.5 ± 1.4 (mean ± SEM; range, 2 to 82) sessions (*n* = 101 session) in the odor task (*n* = 7 rats).

### Fiber photometry

#### Surgery

Wild-type rats (*n* = 11) received bilateral injections of AAV_9_-hSyn-GRAB_DA2m_ (0.8 μl) in the striatum (dNAc; AP: +2.0 mm, ML: ±1.5 mm, DV: +6.1 mm, at an angle of 5°, vlNAc; AP: +0.9 mm, ML: ±2.9 mm, DV: +7.2 mm). Optic fibers (200- or 400-μm core) attached to a 2.5-mm metal ferrule (MFC_200/240-11mm_MF2.5_FLT or MFC_400/430-11mm_MF2.5_FLT, Doric Lenses) were implanted over the striatum (dNAc; AP: +2.0 mm, ML: ±1.5 mm, DV: +5.9 mm, at an angle of 5°, vlNAc; AP: +0.9 mm, ML: ±2.9 mm, DV: +7.0 mm). Recordings were started 4 weeks after virus injections. The laterality of the recording site was counterbalanced across rats both for recording from dNAc (*n* = 7 locations) and vlNAc (*n* = 8 locations). Other signals were excluded from analysis because of fiber misplacement and/or lack of virus expression.

#### Photometry recording

We conducted photometry recording at either unilateral or bilateral locations in the NAc per rat. For simultaneous recording at two locations (*51*), the light from the fiber-coupled light-emitting diode (LED; 470 nm, M470F3, Thorlabs) was collimated (F220SMA-532, Thorlabs) to pass through an excitation filter [MDF-GFP2 (482/18 nm), Thorlabs] and a dichroic mirror (MD416, Thorlabs), reflected by a galvanometer mirror (GVS011, Thorlabs), and a dichroic mirror [MDF-GFP2 (495 nm), Thorlabs] and then focused onto a fiber array of two fiber-optic patch cables (BFYF4LS01, Thorlabs) through an objective lens (numerical aperture 0.50, UPLFNL20X, OLYMPUS). The final output power was adjusted to 8 to 25 μW at the tip of the fiber. The patch cable was connected to two optic fibers (200- or 400-μm core) implanted in the NAc using a ceramic sleeve (ADAF1, Thorlabs). For 405-nm isosbestic excitation (*62*), the light from the fiber-coupled LED (405 nm, M405FP1, Thorlabs) was collimated (F220SMA-532, Thorlabs) to pass through an excitation filter (MF390-18, Thorlabs), reflected by a dichroic mirror (MD416, Thorlabs), the galvanometer mirror, and the dichroic mirror, the same mirrors used for 470-nm light, which was then focused onto the fiber array, as described above. The final output power of 405-nm light was adjusted to approximately match the DA_2m_ fluorescence produced by the 470-nm light. The emission fluorescence was detected using a photomultiplier tube (PMT1001/M, Thorlabs) after passing through the dichroic mirror [MDF-GFP2 (495 nm), Thorlabs] and a filter [MDF-GFP2 (520/28 nm), Thorlabs]. The galvanometer mirror was controlled so that the light alternatively focused on one of the two fibers for 10 ms. LED was then illuminated for 4 ms; Photomultiplier (PMT) signals were recorded at 1 kHz, and the median value during this 4-ms period was used for analysis, which resulted in a 50-Hz signal obtained from each fiber.

For photometry recording at a single location, the galvanometer mirror was focused onto a single fiber, and the LED was continuously illuminated, and PMT signal was recorded at 0.4 kHz. The PMT signals obtained by both methods were low-pass–filtered at 4 Hz. Δ*F*/*F* was calculated as (*F*_a_ − *F*_b_)/*F*_b_, where *F*_a_ was the voltage at any point in time and *F*_b_ was a running median voltage of 100 s. For the analysis of average across animals, baseline normalization (*z* score) was performed on the Δ*F*/*F* for each trial, using 1 s during the ITI as the baseline (Figs. 7, F to I; 8, C to G; and 9, A, C, F, and I to L). The 405-nm control recordings were performed at the end of the recording experiments.

### Calcium imaging

#### Surgery

Two DAT-iCre rats (*31*) received injections of 0.5 μl each of AAV_5_-Syn-Flex-jGCaMP8f or DJ-Syn-Flex-GCaMP7f into three locations of the left VTA (AP: −5.0 mm, ML: 1.6 mm, DV: +7.8 mm, at an angle of 5°; AP: −5.3 mm, ML: 1.6 mm, DV: +7.9 mm, at an angle of 5°; AP: −5.7 mm, ML: 1.4 mm, DV: +8.0 mm, at an angle of 5°). One week after virus injection, we performed tissue aspiration of about 0.8 mm in diameter and a depth of 6.8 mm, using a blunt 23-gauge needle. A GRIN lens (diameter, 1.0 mm; length, 13.7 mm, Inscopix) was then implanted 200 μm above the target area in the VTA (AP: −5.3 mm, ML: 1.6 mm, DV: +7.8 mm, at an angle of 5°). The lens was fixed in place using Kwik-Sil (WPI) and then secured to the skull with a self-curing dental acrylic resin (Unifast II, GC Corporation).

#### Calcium imaging using a miniature microscope

Imaging was conducted after 4 to 6 weeks to allow enough time for virus expression and lens image clearing. Imaging data from DA neurons in the VTA in the auditory task were acquired using nVista (Inscopix) at a frame rate of 10 Hz. The neuronal activities were recorded for 30 min, which corresponded to the duration of one session. Before recording, focal plane, LED power, and gain were optimized individually for each rat.

#### Calcium imaging data processing

Inscopix data processing software (Inscopix) was used to analyze imaging data. First, the data were spatially down sampled by a factor of 4. Then, motion artifacts were corrected using rigid motion correction. After that, the extended constrained nonnegative matrix factorization (*63*), which is optimized for one-photon imaging data, was applied for the data to extract the footprints and fluorescence traces of putative neurons. We manually inspected extracted data and removed non-neural objects. We used the deconvolved fluorescence traces to calculate Δ*F*/*F* as (*F*_a_ − *F*_b_)/*F_b_*, where *F*_a_ was the fluorescence signal at any point in time and *F*_b_ was a running median signal of 100 s.

### Optogenetic experiments

#### Surgery

Fifteen DAT-iCre rats (*31*) received unilateral injections of AAV_5_-EF1α-DIO-ChR2-eYFP (“ChR2 rat”, *n* = 8) or AAV_5_-EF1α-DIO-eYFP (“eYFP rats”, *n* = 7) (0.5 μl) in the striatum (AP: +2.0 mm, ML: ±1.5 mm, DV: +6.1 mm, at an angle of 5°). The laterality of the injections was counterbalanced across rats. Two DAT-iCre rats received bilateral injections of AAV_5_-EF1α-DIO-eYFP. Optic fibers (200-μm core) attached to metal ferrule (MFC_200/240-11mm_MF2.5_FLT, Doric Lenses) were implanted over the striatum (AP: +2.0 mm, ML: ±1.5 mm, DV: +5.9 mm, at an angle of 5°). Optogenetic behavioral testing was conducted 5 weeks after virus injections. Optic fiber implants were connected to a 300-μm-core patch cable (Doric) using a ceramic sleeve, which was connected to a commutator (FRJ_1×1_FC-FC, Doric) by means of an FC/PC adaptor. A second patch cable, with an FC/PC connector at either end, connected the commutator to a 462-nm fiber-coupled laser (BLM462TA, Shanghai Laser & Optics Century).

#### Training before the extinction task

The basic design of a trial was the same as that of the auditory task described above, except for the following. First, after illumination of the cue light, rats could initiate a trial by pushing the lever for 0.4 s, which resulted in delivery of a cue sound (3 kHz, 65 dB, and 0.12 s). Second, in all trials, 0.1% saccharin water (20 μl, 5 μl × 4 shots dispensed every 0.31 s) was delivered. Third, ITI was 0.5 s. Rats were trained to execute 447.7 ± 26.2 trials with 100% reward per session for 6.5 ± 0.6 sessions (mean ± SEM, *n* = 15 rats).

#### Optogenetic behavioral testing in the block extinction task

The basic design of the multiple block extinction task was the same as the training described above, except that rats underwent a 100% reward (R) block and an NR block alternatively in a total of 12 blocks (six each) in a single session. The reward block consisted of 10 trials in which rats could obtain reward by pulling back the lever after the cue sound onset. In the NR block, reward was not presented even after rats pulled the lever. The NR block ended after three total omission trials in which rats failed to pull back the lever closer than lever-position(−1) within 25 s after cue light onset. At the end of an NR block, rats received a free 10-μl reward (5 μl × 2 shots) to encourage them to execute the first trial in the following reward block. In even-numbered NR blocks (i.e., second, fourth, sixth, etc.), rats received optogenetic stimulation (2 s of 20-Hz 10-ms pulsed 462-nm laser, 6 mW), starting 0.4 s after pulling back the lever (same as reward onset in the reward block). Two eYFP rats that received bilateral virus injections underwent two optogenetic tests in 1-week interval and received optogenetic stimulation once on each side. Between the tests, they were trained on reward blocks without optogenetic stimulation for 3 days.

### Histology

Rats were euthanized and perfused transcardially with 4% paraformaldehyde in 0.1 M phosphate buffer under pentobarbital anesthesia (60 mg/kg body weight, intraperitoneally). Brains were sliced at 30-μm thickness using a sliding microtome (Microm HM450, Thermo Fisher Scientific), mounted on glass slides with 4′,6-diamidino-2-phenylindole (DAPI) mounting medium (VECTASHIELD, H-1200, Vector Laboratories), and imaged under a fluorescent microscope (BZ-X700, Keyence).

For immunostaining of tyrosine hydroxylase (TH) in DA neurons (fig. S9E), brain slices were incubated in PBS-T [0.3% Triton X-100 in phosphate-buffered saline (PBS)] with 10% normal goat serum at room temperature for 1 hour. Slices were then incubated with a primary antibody of a rabbit anti-TH (1:500; Millipore, catalog no.AB152, RRID: AB_390204) in PBS-T with 1% normal goat serum for 60 hours at room temperature. After washing with PBS, slices were incubated with a secondary antibody of goat anti-rabbit immunoglobulin G (IgG) antibody conjugated with Alexa Fluor 594 (1:200; Thermo Fisher Scientific, catalog no. A-11012, RRID: AB_2534079) for 16 hours at room temperature. The slices were then mounted on glass slides with a DAPI mounting medium (VECTASHIELD, H-1200, Vector Laboratories) and imaged under a fluorescent microscope (BZ-X700, Keyence).

Double immunostaining of TH and iCre in DA neurons (fig. S2A) was conducted in the same way as above, except that it was performed with a mixture of primary antibodies of a rabbit anti-TH (1:500; Millipore, catalog no. AB152, RRID: AB_390204) and a mouse anti–Cre recombinase (1:500; Millipore, catalog no. MAB3120, RRID: AB_2085748) and, then, a mixture of secondary antibodies of a goat anti-rabbit IgG antibody conjugated with Alexa Fluor 594 (1:200; Thermo Fisher Scientific, catalog no. A-11012, RRID: AB_2534079) and a goat anti-mouse IgG antibody conjugated with Alexa Fluor 488 (1:400; Thermo Fisher Scientific, catalog no. A-11029, RRID: AB_2534088).

### Retrograde labeling

For Retrobeads labeling, rats were injected unilaterally with red fluorescent Retrobeads (50 nl; Red Retrobeads IX, Lumafluor) in dorsal NAc (dNAc; AP: +2.2 mm, ML: ±1.4 mm, DV: +5.4 mm) and were perfused after 2 weeks.

### Data analysis

#### Definition of type 1 and type 2 DA neurons

To classify DA neurons, we compared trial-by-trial activity of each neuron in response 50R versus 50N. Time window was defined as 0.1 to 1.0 s for spiking activity or 0.2 to 2.0 s for calcium levels from R or NR onset. We defined neurons significantly more activated by 50R than 50N as type 1 neurons, and neurons significantly more activated by 50N than 50R as type 2 neurons (two-sided Mann-Whitney *U* test).

#### Definition of the difference of the responses to 50R versus 50N

We first calculated average activity in response to 50R and 50N (time window: 0.1 to 1.0 s for spiking activity or 0.2 to 2.0 s for calcium levels) for each DA neuron. We then quantified the difference in each neuron using the *d*′ measure calculated as

$$d^{\prime }=\frac{\mu_{50R}-\mu_{50N}}{\sqrt{0.5(\sigma_{50R}^{2}+\sigma_{50N}^{2})}}$$

in which μ and σ^2^ are the average and variance of the spiking activity, respectively (Figs. 2F and 5E).

#### Event-related spiking activity analysis

To examine spiking rates, peristimulus time histograms (PSTHs) were constructed using 50-ms (Figs. 2, A to D; and 3, A, C, F; and 4A and figs. S3 and S4A) or 10-ms (Fig. 4, E and H, and fig. S4, E, H, and K) bins, *z*-scored, and smoothed using a Gaussian kernel (σ = 1.5 bins). *Z* scores were calculated as (*x* − *b*)/SD, where *x* is the mean spiking rate in each bin, *b* is the mean spiking rate during the baseline period (1 s during ITI), and SD is the SD of the baseline period.

#### Definition of activity modulation by expected reward probability

For each neuron, we calculated average spiking rate during 0.3 to 0.8 s after cue onset. We then examined which cue most activated the response (Fig. 3E).

#### Comparison of the activities between early versus late responses to reward delivery

For each neuron, we first calculated average spiking activity of early (blue bar in Fig. 4A and fig. S4A: 0 to 1 s for type 1 and 0.4 to 1.4 s for type 2) and late (gray bar in Fig. 4A and fig. S4A: 1 s before the last shot of reward) responses to reward delivery across all trials. We then compared the activities across all type 1 or type 2 neurons (Fig. 4B and fig. S4B).

#### Comparison of the activities before versus after reward termination

We first calculated average spiking activity of each neuron across all trials before (4 to 5 s after reward onset) and after (5 to 6 s after reward onset) reward end. We then quantified the difference in each neuron using the *d*′ measure calculated as

$$d^{\prime }=\frac{\mu_{\mathrm{after}}-\mu_{\mathrm{before}}}{\sqrt{0.5(\sigma_{\mathrm{after}}^{2}+\sigma_{\mathrm{before}}^{2})}}$$

in which μ and σ^2^ are the average and variance of the spiking activity, respectively. Negative or positive d′ values represent activity consistent with RPE or type 2–like signal, respectively. To evaluate whether RPE or type 2–like signal was significantly represented across the neurons (Fig. 4, C and D, and fig. S4, C and D), we examined whether the *d*′ distribution was significantly different from zero using a two-sided Wilcoxon signed-rank test (Fig. 4D and fig. S4D).

#### Trial-by-trial variability of spiking rates

We construct a PSTH for each unit aligned to reward (or NR) onset using 10-ms bins. We then determined which 10-ms bin is the positive (or negative) peak. Time window to find the positive (or negative) peak was from 0.1 to 1.2 s after R/NR onset. We next calculated trial-by-trial spiking rate during 0.5 s around the peak (±0.25 s from the peak) and subtracted baseline activity (1 s during ITI). We then calculated SD of the spiking rates across the trials and plotted them across all neurons (Fig. 4K).

#### Correlation analysis between behavior and neural activity

To examine correlation between NR-Push(1) latency and spiking activity (or DA level) (Figs. 6 and 7 and fig. S6), we focused on trials in which rats responded relatively quickly and continued to engage. For this, we selected trials based on four criteria (see table S1 for the percentages of neurons that passed each criterion and all criteria). First, trials in which rats released the lever after pulling it closer than the lever-position(−1) were excluded. Second, Cue-Pull latency was less than 1.5 s. Third, NR-Push(1) latency was shorter than 5.6 s (6.4 s for DA level), which was the shortest time to proceed to the next trial. Fourth, outlier values, defined as values more than 1.5 interquartile ranges above the upper quartile (75%) or below the lower quartile (25%), were excluded. Almost all of the outlier values were above the upper quartile.

To examine the correlation between Push(−1)-Push(1) latency and spiking activity (Fig. 6 and figs. S5 and S6), we selected trials based on the same criteria with those for NR-Push(1) latency. The third criterion was that NR (or reward end)–Push(1) latency was shorter than 5.6 (or 6.0) s. We excluded the neurons with less than eight passed trials in either condition from the following analysis. The number of the excluded neurons were three or five neurons for type 1 or type 2 neurons, respectively.

Using the selected trials of the selected neurons (*n* = 78 or 73 neurons for type 1 or type 2 neurons, respectively), we next construct a PSTH for each neuron aligned to NR or the time crossing Push(−1). Neural activity was averaged in 100-ms bins, shifted by 1 ms (100 bins, centered on current bin). We then determined which 1-ms bin is the positive peak for type 2 (or negative peak for type 1). Time window to find the peak was from 0.2 to 1.2 s after NR onset or −0.5 to 0.5 s after the time crossing Push(−1). Trial-by-trial spiking rate during −0.1 to 0.4 s (or −0.4 to 0.1 s) from the peak was calculated, and baseline activity (1 s during ITI) was subtracted. We then conducted trial-by-trial correlation analysis (Spearman’s rank correlation test) between the activity and NR-Push(1) [or Push(−1)-Push(1)] latency. For the correlation analysis in Fig. 6G and fig. S6J, trials in which Cue-Pull latency was less than 1.5 s were analyzed. A total of 5.5% of the trials were excluded. Trial-by-trial spiking rate during 0.2 to 0.5 s from cue onset was calculated, and baseline activity (1 s during ITI) was subtracted. We then conducted a trial-by-trial correlation analysis (Spearman’s rank correlation test) between the activity and Cue-Pull latency.

#### Analysis of DA levels in the Pavlovian task

Numbers of the trials used for the analysis were as follows: Fig. 8C (top), “Before”: *n* = 637 or 321 total trials in seven rats for 100R or 50R, respectively; from Pavlovian “Day 1” to “Last”: *n* (range) = 206 to 219 or 103 to 109 total trials in seven rats, respectively; Fig. 8C (bottom), Before: *n* = 625 or 307 total trials in eight rats for 100R or 50R, respectively; from Pavlovian Day 1 to Last: *n* (range) = 241 to 257 or 121 to 126 total trials in eight rats, respectively; Fig. 8F (top), Before: *n* = 318 or 450 total trials in seven rats for 50N or 0N, respectively; from Pavlovian Day 1 to Last: *n* (range) = 103 to 108 or 205 to 218 total trials in seven rats, respectively; and Fig. 8F (bottom), Before: *n* = 308 or 406 total trials in eight rats, respectively; from Pavlovian Day 1 to Last: *n* (range) = 124 to 130 or 249 to 256 total trials in eight rats, respectively.

Time windows used to analyze responses to reward (Fig. 8C, light blue bar) or responses after reward termination (Fig. 8C, gray bar) are 0.2 to 1.2 s for dNAc and 0 to 1 s for vlNAc or 4.5 to 5.5 s for dNAc and 3.5 to 4.5 s for vlNAc, respectively. Time windows used to analyze responses to NR (Fig. 8F, magenta bar) are 1.5 to 3 s for dNAc and 0.5 to 2 s for vlNAc.

#### Correlation between licking behavior and DA levels in the Pavlovian task

In rewarded trials in the Pavlovian conditioning (Figs. 8 and 9), we define the last lick after reward end as a lick after which the next lick was more than 0.4 s later and then defined the latency to stop licking as latency from the reward end to the last lick (Fig. 9, G and J). For the number of licks used for the correlation analysis (fig. S8, H, I, and K), we measured the number during 3.5 to 4.5 s (for dNAc) or 3.0 to 4.0 s (for vlNAc) from reward onset. We used all trials for the correlation analysis. In no-rewarded trials, we define the last lick after cue end as a lick after which the next lick was more than 1 s later and then defined the latency to stop licking as latency from the cue end to the last lick (Fig. 9, K and L). We used trials (55.1%) with at least one lick within 3 s after the end of cue 2. Because the number of licks after NR was very small, we did not use it for the correlation analysis.

#### Behavioral analysis in the optogenetic experiment

For Cue-Pull latency and NR-Push(1) latency, outlier values, defined as values more than 1.5 interquartile ranges above the upper quartile (75%) or below the lower quartile (25%) among those in the first six nonreward blocks, were excluded from the analysis. This was necessary because rats were allowed to spend ~25 s, which was much longer than average latency, to pull back the lever. As a result, 86.5% of the trials were used for Cue-Pull latency (fig. S9, O and P), and 90.5% of the trials were used for NR-Push(1) latency (fig. S9, I and J). Almost all of the outlier values were above the upper quartile. The trial with an outlier of NR-Push(1) latency was also excluded from the analysis of NR-Push(−1) latency and Push(−1)-Push(1) latency (fig. S9, K to N).

### Statistical analyses and data presentation

All data are expressed as means ± SEM unless stated otherwise. In box plots, the central mark and the edges represent the median and the 25th and 75th percentiles, respectively. The whiskers represent the data range. In many of the plots, outlier values are not shown for visualization purpose. However, all data points and animals were included in all the statistical analysis.

## Notes

View/request a protocol for this paper from *Bio-protocol*.
