# Supplementary material for: Dopamine error signal to actively cope with lack of expected reward
Source: Sci Adv. 2023 Mar 10;9(10):eade5420. doi: 10.1126/sciadv.ade5420 (PMC10005178; doi:10.1126/sciadv.ade5420)
Supplement: Supplementary file 1 — Supplementary Text Figs. S1 to S9 Table S1 [file sciadv.ade5420_sm.pdf]

Science Advances
AAAS

Supplementary Materials for

Dopamine error signal to actively cope with lack of expected reward

Seiya Ishino *et al.*

Corresponding author: Masaaki Ogawa, ogawa.masaaki.7m@kyoto-u.ac.jp

Sci. Adv. **9**, eade5420 (2023)
DOI: 10.1126/sciadv.ade5420

This PDF file includes:

Supplementary Text
Figs. S1 to S9
Table S1

Supplementary Text

Causal relevance of type 2 DA error signal in dNAc and behavioural adjustment to actively cope with lack of expected reward (fig. S9)

To test the possibility that the type 2 DA error signal transmitted to dNAc causally relates to behavioural adjustment to actively cope with lack of expected reward, we introduced a multiple block extinction task, combined with optogenetic activation of DA inputs to dNAc (fig. S9, A to D). In this task, blocks of reward or no-reward trials were alternated multiple times (six times each) in a single session, such that reward expectation in the extinction block would gradually decrease to zero. We omitted reward in the no-reward blocks, and rats were allowed to go to the next reward block after three "no-go" trials in which rats essentially gave up moving on to the next trial (i.e., not pulling the lever within 25 s after cue light onset).

To enable optogenetic activation of DA inputs to dNAc, we expressed either ChR2-eYFP ("ChR2 rats") or eYFP ("eYFP rats") in DA neurons projecting to dNAc (fig. S9E), and stimulated ChR2 (or eYFP only)-expressing dopaminergic axons in dNAc (fig. S9F) at the time of reward omission in even-numbered no-reward blocks (i.e., 2nd, 4th, etc.; fig. S9C). Before the extinction session, rats were trained to execute 447.7 ± 26.2 trials with 100% reward per session for 6.5 ± 0.6 sessions (mean ± s.e.m., n = 15 rats). In the extinction session, as rats were exposed to more no-reward blocks, the number of trials executed in each block gradually decreased both in ChR2 and eYFP rats (fig. S9, G and H). However, in the early half of the no-reward blocks ("First 6", fig. S9H), ChR2 rats executed more trials in the blocks with the optogenetic activation than in those without the activation. This increase was neither evident in the latter half of the no-reward blocks, nor was it observed in eYFP rats (fig. S9G). Further, NR-Push(1) latency, but not latency from no-reward onset to push the lever toward the middle range ["NR-Push(-1) latency"], became longer across the early no-reward blocks in eYFP rats (fig. S9, I, K, M, and O), consistent with suppressed switching toward the next reward after no-reward. This increase in NR-Push(1) latency was not observed in ChR2 rats (fig. S9, J , L, N, and P).

These results show that the optogenetic activation of DA inputs to dNAc causally relates to behavioral adjustment toward the next reward after no-reward as long as the rats are still expecting the next reward, although we do not exclude the possibility that RPE-type DA inputs could, in part, contribute to the behavioral effects (*37*).

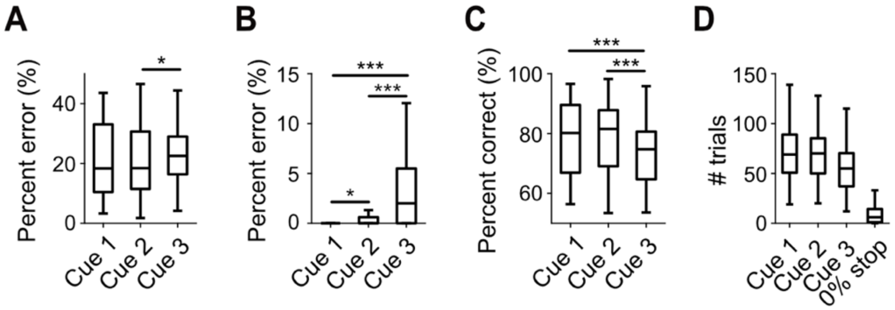

Fig. S1. Error trials in the operant cue-reward association task.
(**A** and **B**) Percentage of error trials in the operant cue-reward association task (as in Fig. 1, A to E) in which rats pulled the lever before the offset of odor cues (A), or rats did not pull the lever within 1.5 s after the odor offset (B). Significant difference between conditions, $n = 101$ sessions across 7 rats, $*P < 0.05$, $***P < 0.001$, two-sided Wilcoxon signed-rank test with Bonferroni correction. (**C**) Percentage of "correct" trials defined as total trials minus the sum of the two error trials [as in (A and B)]. $***P < 0.001$, two-sided Wilcoxon signed-rank test with Bonferroni correction. (**D**) Number of trials for each trial type across recording sessions. "0% stop", trials in which rats did not pull the lever closer than the lever-position(-1) before cue light was turned off (see Methods for the details). $n = 101$ sessions.

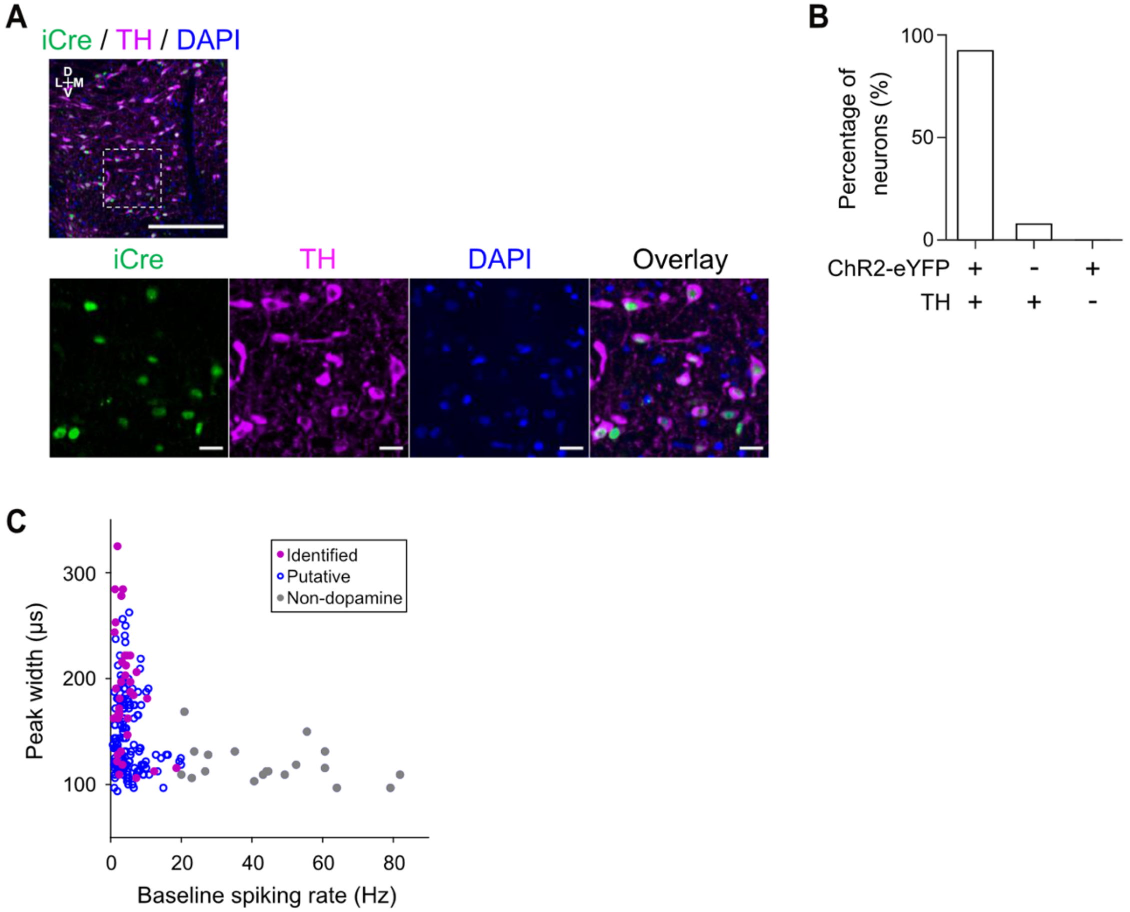

Fig. S2. Histological verification of optogenetic identification of DA neurons.
(**A**) Top: immunostaining for iCre (green), TH (DA neurons, magenta), stain for DAPI (cell nuclei, blue), and their overlay in the left VTA in a DAT-iCre rat. Scale bar: 500 µm. Bottom: magnified view of the area inside the white-dashed rectangle in top. Scale bars: 20 µm. All of the iCre-positive cells were TH-positive. (**B**) Specific and efficient expression of ChR2-eYFP in DA neurons. Percentage of neurons labeled for ChR2-eYFP and TH in the lateral VTA in a DAT-iCre rat injected with AAV- EF1α-DIO-ChR2-eYFP in VTA. n = 129 neurons in 2 coronal brain slices (AP: -5.2 mm and -5.7 mm). See fig. S9E for example immunostaining. (**C**) Baseline spiking rate versus spike width at half-maximum for each DA neuron. Non-DA neurons were analyzed only when they were recorded simultaneously with identified and/or putative DA neurons.

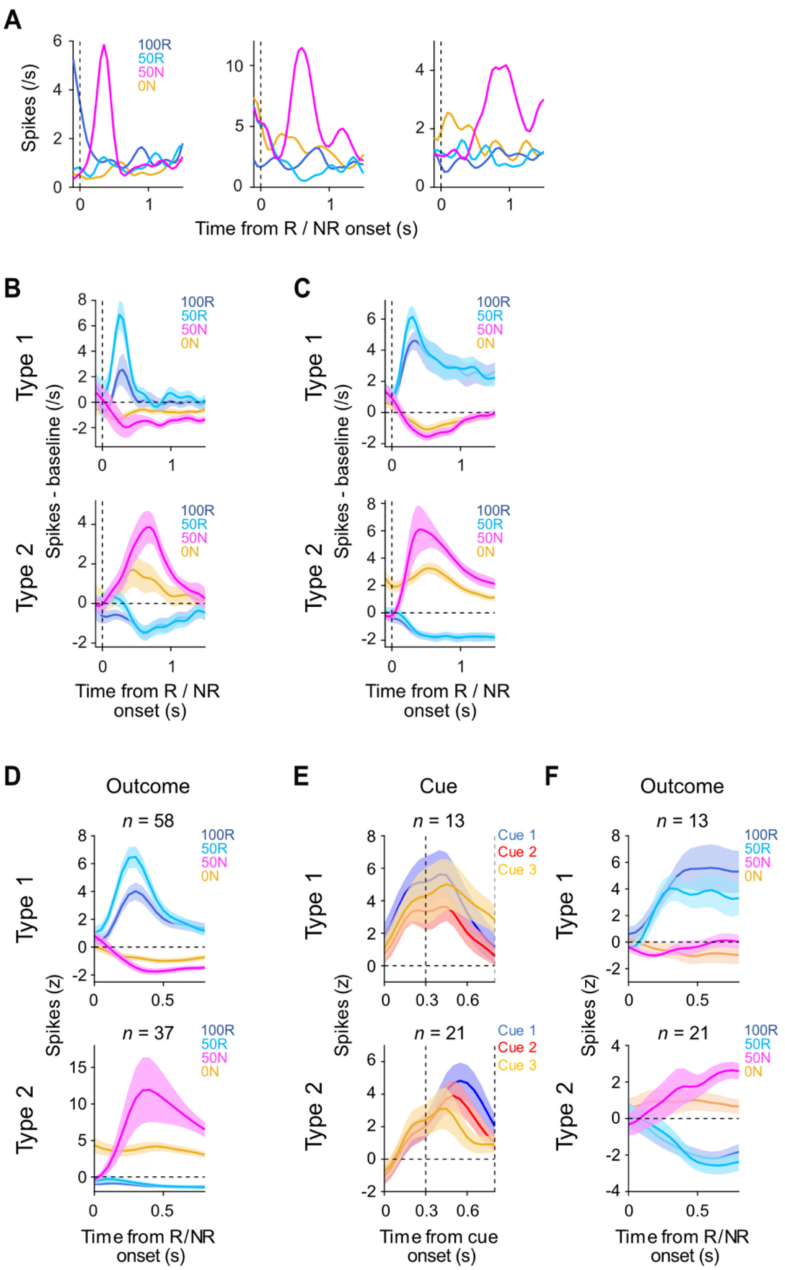

Fig. S3. Spiking activity of identified and putative DA neurons in response to reward outcomes and cues.

(**A**) Responses of three example optogenetically-identified DA neurons (shorter peak latency in response to 50N from the left) to reward outcomes. (**B**) Baseline subtracted average spiking rates of identified type 1 (top) or type 2 (bottom) neurons (the same neurons as in Fig. 3A). (**C**) Same as (B), but for putative DA neurons (the same neurons as in Fig. 3C). (**D**) Responses of type 1 (top, *n* = 58) or type 2 (bottom, *n* = 37) neurons (as in Fig. 3F) during outcome periods. (**E**) Same as Fig. 3F, but for type 1 neurons that were most activated by cue 3 (top, *n* = 13) or type 2 neurons that were most activated by cue 1 (bottom, *n* = 21). (**F**) Responses of the type 1 (top) or type 2 (bottom) neurons [as in (E)] during outcome periods.

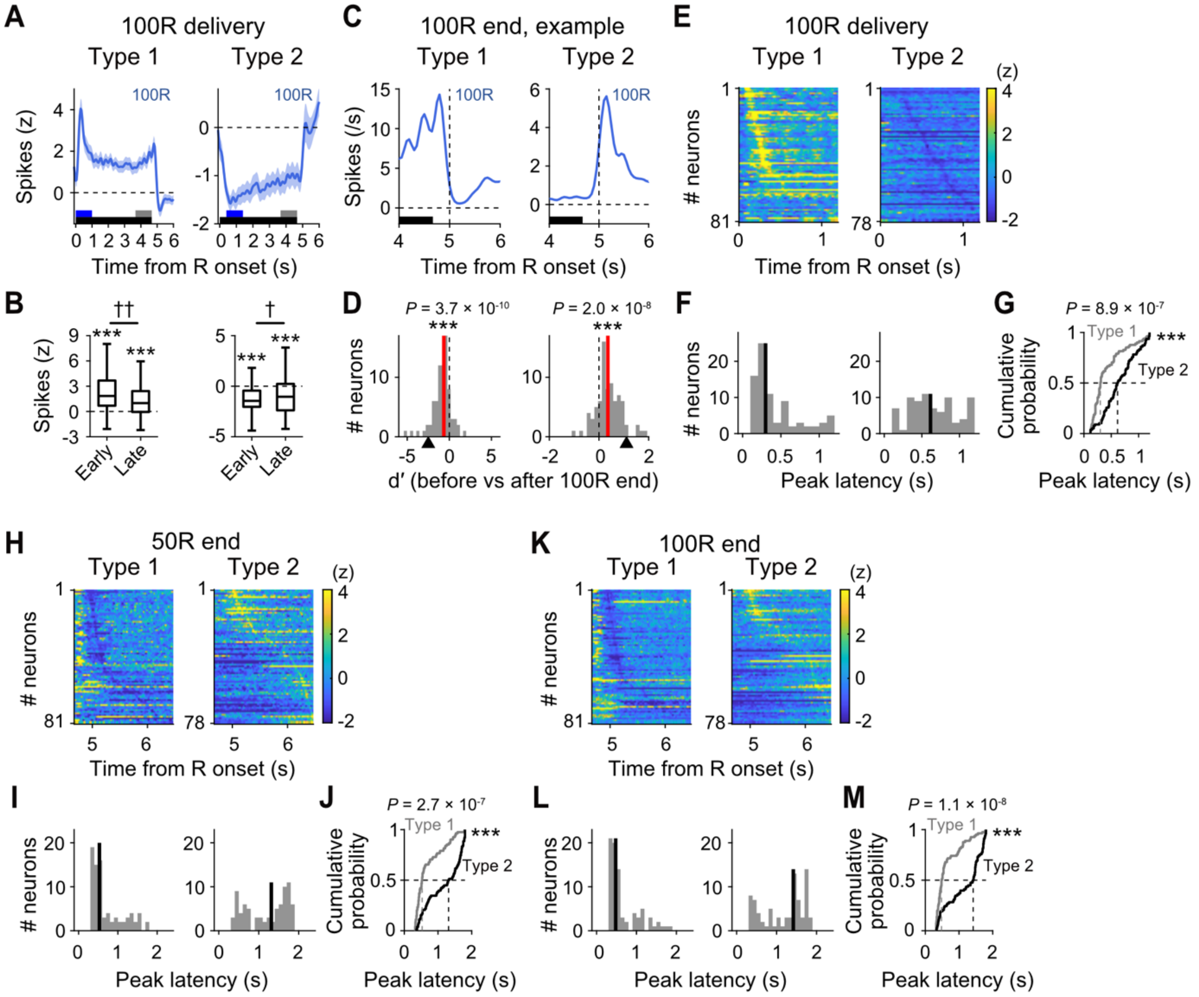

Fig. S4. Spiking activity of all DA neurons during reward delivery and reward end.
(**A** and **B**) Same as Fig. 4, A and B, but for 100R delivery. Significant difference from baseline, ***$P < 0.001$, two-sided Wilcoxon signed-rank test (B). Significant difference between conditions, †$P < 0.05$, ††$P < 0.01$, two-sided Wilcoxon signed-rank test (B). (**C** and **D**) Same as Fig. 4, C and D, but for 100R end. Significant shift from zero, ***$P = 3.7 \times 10^{-10}$ (left); $P = 2.0 \times 10^{-8}$ (right), two-sided Wilcoxon signed-rank test (D). (**E** to **G**) Same as Fig. 4, H to J, but for 100R. ***$P = 8.9 \times 10^{-7}$, Kolmogorov–Smirnov test (G). (**H** to **J** and **K** to **M**) Same as (E to G)**,** but for 50R or 100R end, respectively. Peak latency is the latency from the last shot of reward delivery (4.7 s after reward onset). ***$P = 2.7 \times 10^{-7}$ (J) and ***$P = 1.1 \times 10^{-8}$ (M).

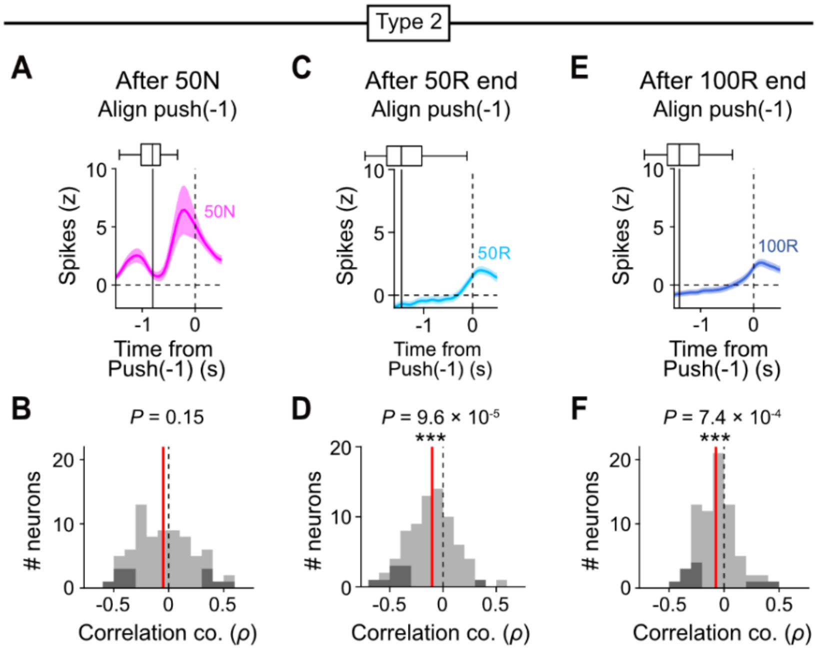

Fig. S5. Correlation between spiking activity of type 2 DA neurons and behavioural switching toward the next reward.

(**A** and **B**) Same as Fig. 6, D, top, and F, but after 50N. $P = 0.15$ (B). (**C**) Same as Fig. 6D, top, but for activity of type 2 neurons aligned to the time crossing the lever-position(-1) after the end (last shot) of 50R. Box plot, average onsets of the last shot of reward in the recording sessions. (**D**) Same as Fig. 6F, but for correlation coefficients between spiking activities after the end of 50R and Push(-1)-Push(1) latencies across all type 2 neurons. ***$P = 9.6 \times 10^{-5}$. (**E** and **F**) Same as (C and D), but after 100R end. ***$P = 7.4 \times 10^{-4}$ (F).

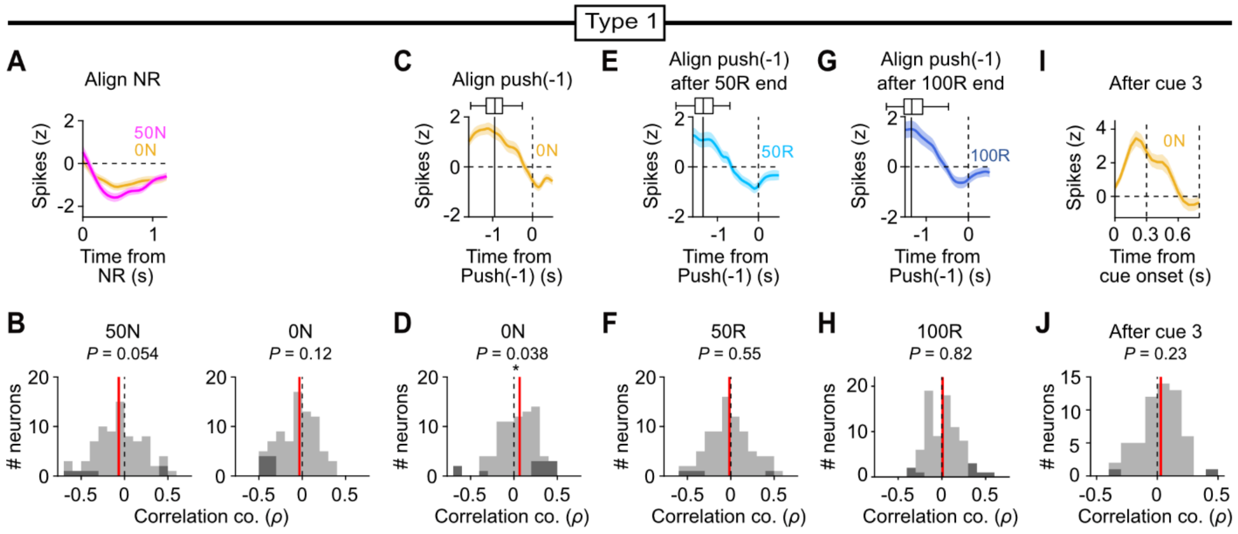

Fig. S6. Correlation between spiking activity of type 1 DA neurons and behavioural switching toward the next reward.

(**A** and **B**) Same as Fig. 6, A and C**,** but for type 1 neurons (n = 78 neurons). P = 0.054 after 50N; P = 0.12 after 0N (B). (**C** and **D**) Same as Fig. 6, D and F**,** but for type 1 neurons. *P = 0.038 (D). (**E** and **F**) Same as fig. S5, C and D**,** but for type 1 neurons. P = 0.55 (F). (**G** and **H**) Same as (E and F), but after 100R end. ***P = 0.82 (H). (**I** and **J**) Same as Fig. 6G**,** but for type 1 neurons. P = 0.23 (J).

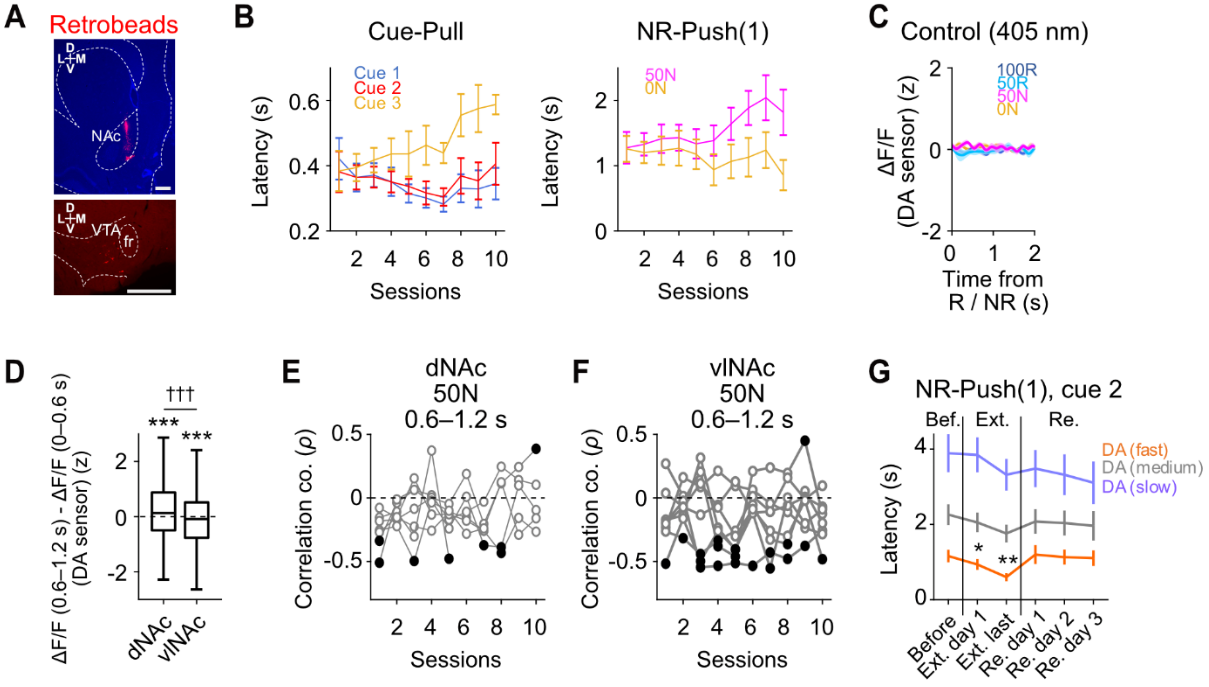

Fig. S7. Behaviours and DA levels in the NAc during initial training, the 50% reward extinction, and the 50% reintroduction.

(**A**) Retrobeads injected in dNAc (top, red) transported retrogradely to anterior lateral VTA (bottom). Note many beads-positive neurons in lateral VTA. fr: fasciculus retroflexus. Scale bar: 0.5 mm. (**B**) Cue-Pull (left) or NR-Push(1) (right) latencies across early sessions in the operant cue-association task (n = 11 rats). Mean ± s.e.m. (**C**) 405-nm control signals in NAc (n = 11 rats in total; n = 4 in dNAc and n = 7 in vlNAc). (**D**) Trial-by-trial difference of DA levels during 0.6–1.2 s and those during 0–0.6 s in dNAc or vlNAc after 50N (sessions 8–10). Significant difference from baseline, ***$P < 0.001$, two-sided Wilcoxon signed-rank test. Significant difference between locations, †††$P < 0.001$, two-sided Mann-Whitney U test. (**E**) Correlation coefficient between DA level in dNAc during 0.6–1.2 s and NR-Push(1) latency across the sessions. Each point represents each rat in each session. Black point indicates significant correlation coefficient (P < 0.05, Spearman's rank correlation test). (**F**) Same as (E), but for DA levels in vlNAc. (**G**) NR-Push(1) latencies after cue 2 divided into fast (25% of all trials, orange), medium (50%, gray), and slow (25%, purple) latencies across the task in which 50R after cue 2 was first extinguished and then reintroduced. Mean ± s.e.m. Significant difference of latency as compared to that before extinction. *$P < 0.05$, **$P < 0.01$, two-sided Wilcoxon signed-rank test with Bonferroni correction.

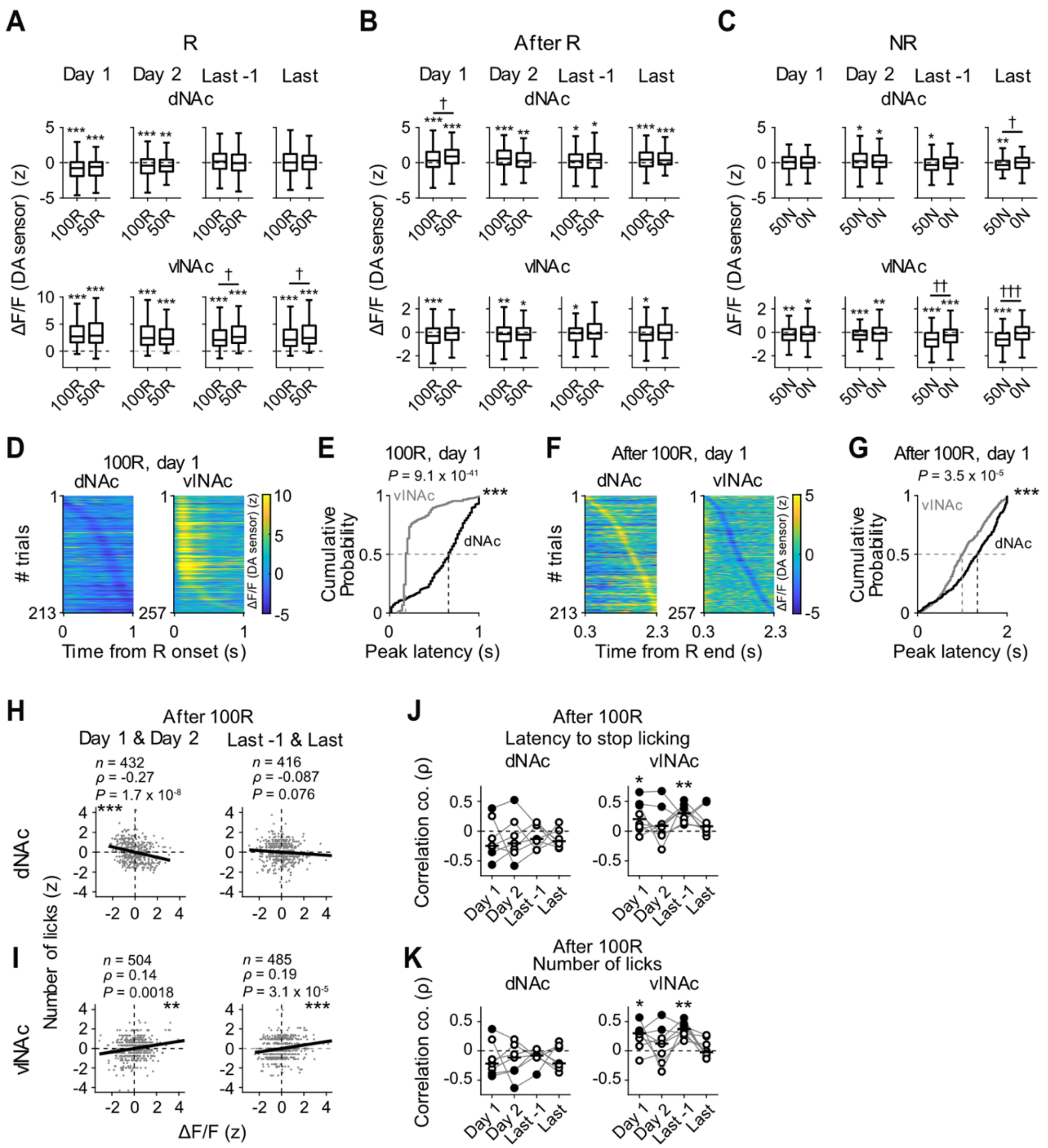

Fig. S8. Detailed analysis of DA responses in the NAc in the transition from the operant to the Pavlovian task.

(**A** to **C**) Same as Fig. 8, D, E, and G (right), but box plots for comparisons in all days. (**D** and **E**) Same as Fig. 9, A and B, but for 100R. ***$P = 9.1 \times 10^{-41}$, Kolmogorov–Smirnov test (E). (**F** and **G**) Same as Fig. 9, C and D, but for after 100R. ***$P = 3.5 \times 10^{-5}$, Kolmogorov–Smirnov test (G). (**H** and **I**) Same as Fig. 9, G and J, but for number of licks. See Methods for the definition of number of licks. (**J**) Correlation coefficient between latencies to stop licking and DA levels in dNAc (left) or vlNAc (right) across all sessions. Each point represents the correlation coefficient in each rat. Black point indicates significant correlation coefficient, respectively ($P < 0.05$, Spearman's rank correlation test). Significant difference from baseline across rats, *$P < 0.05$; **$P < 0.01$, Wilcoxon's singed-rank test. (**K**) Same as (J), but for number of licks.

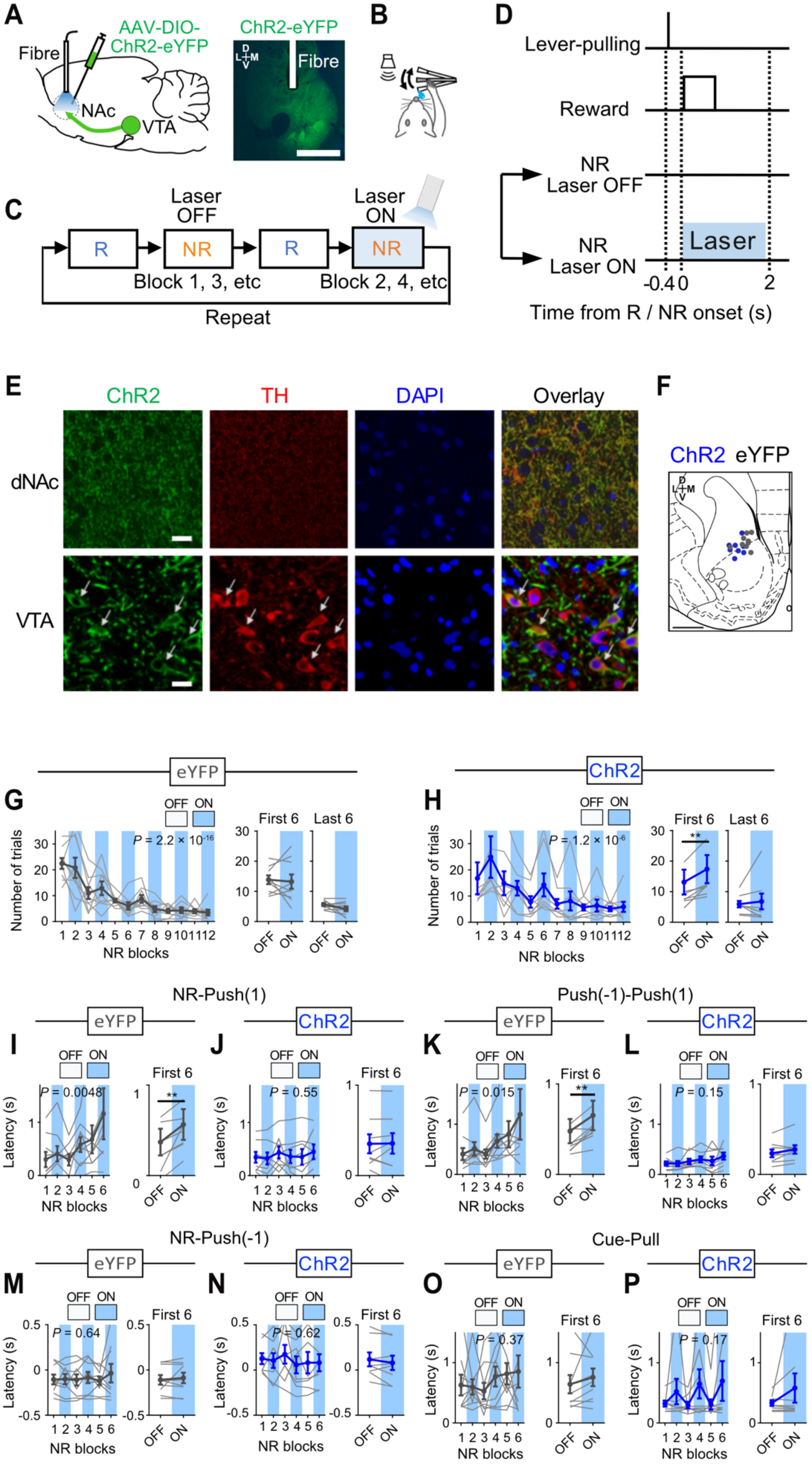
A
Fibre
AAV-DIO-
ChR2-eYFP
NAc
VTA
ChR2-eYFP
Fibre
B
C
Laser
OFF
Laser
ON
R
NR
R
NR
Block 1, 3, etc
Block 2, 4, etc
Repeat
D
Lever-pulling
Reward
NR
Laser OFF
NR
Laser ON
Laser
-0.4
0
2
Time from R / NR onset (s)
E
ChR2
TH
DAPI
Overlay
dNAc
VTA
F
ChR2 eYFP
eYFP
G
OFF ON
P = 2.2 × 10-16
Number of trials
NR blocks
First 6
Last 6
OFF ON
ChR2
H
P = 1.2 × 10-6
Number of trials
NR blocks
First 6
Last 6
NR-Push(1)
Push(-1)-Push(1)
I
P = 0.0048
Latency (s)
NR blocks
First 6
J
P = 0.55
K
P = 0.015
L
P = 0.15
NR-Push(-1)
Cue-Pull
M
P = 0.64
N
P = 0.62
O
P = 0.37
P
P = 0.17

Fig. S9. Causal relevance of type 2 DA error signal in dNAc and behavioural adjustment to actively cope with lack of expected reward

(**A**) Left: schematic of optogenetic activation. Right: example ChR2-eYFP expression in dopaminergic axons (green) and optic fibre placement (white) in dNAc. Scale bar: 1 mm. (**B**) Schematic of the auditory task. A single auditory cue was associated with reward in all trials in a reward block, or with no-reward in a no-reward block. (**C**) Sequence of reward (R) and no-reward (NR) blocks in the block extinction task. Laser was activated in every other NR block ("Laser OFF" or "Laser ON"). (**D**) Timing of the optogenetic activation (blue bar, 2 s, "Laser ON"). (**E**) Expression of ChR2-eYFP (green) with immunostaining for TH (DA neurons, red), stain for DAPI (cell nuclei, blue), and their overlay in dNAc (top) and VTA (bottom) in a DAT-iCre rat used for optogenetic stimulation of DA axons in dNAc. Scale bars: 20 μm. Arrows indicate ChR2-expressing DA neurons. (**F**) Histological verification of approximate location of the tip of each optic fibre in dNAc (AP: +2.2 mm). Scale bar: 1 mm. Purple, ChR2 rats (n = 8); Gray, eYFP rats (n = 9). (**G**) Left: average number of the trials in which eYFP rats (n = 9) pulled back the lever in each NR block. Light blue, Laser ON block. Gray circle, average across rats; error bar, s.e.m. Thin gray line, individual rat. One-way repeated measures ANOVA, F (1,11) = 16.6, $P = 2.2 \times 10^{-16}$. Right: average number of the trials in Laser OFF ("OFF") versus Laser ON ("ON") condition across the first (left) or the last (right) six NR blocks [as in (G), left]. For example, "OFF" in "First 6" indicates the 1st, 3rd and 5th NR blocks, whereas "ON" indicates the 2nd, 4th and 6th blocks. Two-sided Wilcoxon signed-rank test, first 6, P = 0.89 (left); last 6, P = 0.23 (right). (**H**) Left: same as in (G)**,** but for ChR2 rats (n = 8). F (1,11) = 5.7, $P = 1.2 \times 10^{-6}$. Right: first 6, **P = 0.0078 (left); last 6, P = 0.92 (right). (**I**) Left: average NR-Push(1) latency across eYFP rats in each NR block. One-way repeated measures ANOVA, F (1,5) = 4.0, P = 0.0048. Right: average NR-Push(1) latency across the first six NR blocks (as in left). Two-sided Wilcoxon signed-rank test, **P = 0.0039. (**J**) Same as in (I)**,** but for ChR2 rats. Left: F (1,5) = 0.81, P = 0.55. Right: P = 0.95. (**K**) Same as (I), but for Push(-1)-Push(1) latency. Left: F (1,5) = 3.3, *P = 0.015, one-way repeated measures ANOVA. Right: **P = 0.0078, two-sided Wilcoxon signed-rank test. (**L**) Same as (K), but for ChR2 rats. Left: F (1,5) = 1.8, P = 0.15, one-way repeated measures ANOVA. Right: P = 0.11, two-sided Wilcoxon signed-rank test. (**M**) Same as (I), but for NR-Push(-1) latency. Left: F (1,5) = 0.68, P = 0.64, one-way repeated measures ANOVA. Right: P = 0.91, two-sided Wilcoxon signed-rank test. (**N**) Same as (M), but for ChR2 rats. Left: F (1,5) = 0.71, P = 0.62, one-way repeated measures ANOVA. Right: P = 0.31, two-sided Wilcoxon signed-rank test. (**O**) Same as (I), but for Cue-Pull latency. Left: F (1,5) = 1.1, P = 0.37, one-way repeated measures ANOVA. Right: P = 0.36, two-sided Wilcoxon signed-rank test. (**P**) Same as (O), but for ChR2 rats. Left: F (1,5) = 1.6, P = 0.17, one-way repeated measures ANOVA. Right: P = 1, two-sided Wilcoxon signed-rank test.

Table S1. Percentage of trials that met each criterion for the correlation analysis between each behavioural latency and neural activity.

Spiking activity

NR-Push(1) latency (Fig. 4, A, B, G, and H)

	Criterion	50N	0N
1	Lever release	93.7	88.0
2	Cue-Pull < 1.5 s	99.7	94.1
3	NR-Push(1) < 5.6 s	92.0	96.1
4	Outlier	94.4	94.8
	All criteria	80.5	74.9

Push(-1)-Push(1) latency (Fig. 4, C to F, I to L, and fig. S5, A to F)

	Criterion	100R end	50R end	50N	0N
1	Lever release	94.4	94.0	93.7	88.0
2	Cue-Pull < 1.5 s	99.7	99.6	99.7	94.1
3	NR-Push(1) < 5.6 s (6.0 s for reward end)	94.0	93.7	92.0	96.1
4	Outlier	96.4	96.4	95.0	93.9
	All criteria	84.6	83.8	81.1	74.0

DA levels

NR-Push(1) latency (after cue 2) (Fig. 5, F to I)

	Criterion	Before	Ext. day 1	Ext. day 2	Re. day 1	Re. day 2	Re. day 3
1	Lever release	96.9	96.0	97.4	96.3	96.0	99.0
2	Cue-Pull < 1.5 s	96.0	97.4	92.6	86.2	96.7	98.0
3	NR-Push(1) < 6.4 s	94.9	96.0	97.5	98.2	97.1	97.3
4	Outlier	93.1	95.0	96.9	93.3	94.5	95.5
	All criteria	85.3	87.1	85.6	76.5	85.3	91.2
